# Respiratory Protective Effects of Perilla Leave Varieties (*Perilla frutescens*) Against Fine Particulate Matter (PM_2.5_ )‐induced Damage in Human Nasal Cells

**DOI:** 10.1002/fsn3.4708

**Published:** 2024-12-22

**Authors:** Min Young Kim, Jung In Kim, Sang Woo Kim, Sungup Kim, Eunyoung Oh, Jeongeun Lee, Eunsoo Lee, Yeon Ju An, Chae‐Yeon Han, Heungsu Lee, Myoung Hee Lee

**Affiliations:** ^1^ Department of Southern Area Crop Science National Institute of Crop Science, Rural Development Administration Milyang Korea

**Keywords:** fine particulate matter, genetic resource, Perilla leaf, respiratory disease, varieties

## Abstract

Fine particulate matter (PM2.5) is known to exacerbate chronic respiratory disorders, primarily by inducing inflammatory responses and mucus overproduction. Perilla leaves are reported to have significant health benefits, such as antioxidant, antibacterial, and antiallergic properties, attributed to phenolic compounds that vary depending on genetic diversity. In this study, flavonoid‐rich extracts (FRE) from 56 perilla leaf varieties and genetic resources were prepared and screened using a mass screening system. The screening focused on evaluating their anti‐inflammatory, mucus‐reducing, and respiratory protective effects against PM2.5‐induced damage in human nasal cells (RPMI2650). Parameters such as cell viability, nitric oxide (NO) levels, and mucus secretion factor (MUC5AC) concentrations were assessed. Among the 56 varieties, *Perilla frutescens var. crispa* (YCPL706), sourced from Ulleung Island, Korea, exhibited the highest cell viability (112.50%, 100 μg/mL), lowest NO concentration (9.98 μM, 100 μg/mL), and MUC5AC level (78.65 ng/mL, 100 μg/mL). Further evaluation of YCPL706 FRE demonstrated significant respiratory protective effects, including the inhibition of pro‐inflammatory cytokines (TNF‐α, IL‐6, and IL‐1β), MUC5AC, and oxidative stress factors (MDA and ROS), compared to the control cultivar Namcheon. YCPL706 also showed strong antibacterial activity against *Pseudomonas aeruginosa* (minimum inhibitory concentration: 5 mg/mL). These findings suggest that the genetic resource YCPL706 is a promising candidate for combating PM2.5‐induced respiratory damage due to its potent anti‐inflammatory, antioxidant, and antibacterial properties.

## Graphical abstract

This study demonstrates the potential of perilla leaves as a protective agent against PM2.5‐induced respiratory damage, highlighting genetic diversity's role in phytochemical accumulation. Among 56 varieties, the selected resource (YCPL706) protects the respiratory system by reducing oxidative stress, inflammation, mucus hypersecretion, and fibrosis, thereby preventing respiratory diseases induced by pathogenic bacteria, such as *P. aeruginosa* .

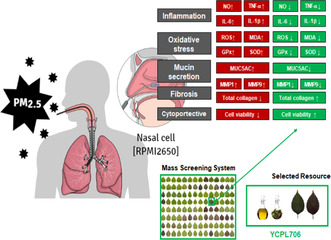

## 1 Introduction

Ambient fine particulate matter, or PM_2.5_, is a serious health threat to individuals worldwide (Atkinson et al. 2014). According to the 2017 Global Burden of Disease Study, PM_2.5_ causes 4.58 million premature deaths per year, 1.12 million of which occur in China (GBD 2017 Risk Factor Collaborators 2018). PM2.5 exposure has been linked to various diseases, including chronic obstructive pulmonary disease (COPD), asthma, cardiovascular diseases, and lung cancer (Pope and Dockery 2006). Although the exact molecular mechanisms by which PM_2.5_ negatively impacts human health remain unknown, oxidative stress and inflammatory responses induced by PM_2.5_ exposures are considered important factors (Ramalingam and Rajaram 2021). Transition metals, specifically those with a high acid content, may disrupt the defense processes of the host and promote inflammation (Song, You, and Chen 2023). In vitro studies on PM_2.5_ exposures have reported the occurrence of cytotoxicity, oxygen radical formation, and cytokine release, suggesting an association with inflammatory disorders (Platel et al. 2020). Han et al. (2022) investigated the relationship between PM_2.5_ inhalation and nuclear factor kappa B (NF‐κB)‐related inflammation in pulmonary epithelial cells. Fine dust can activate inflammatory pathways through reactive oxygen species (ROS)‐dependent mechanisms (Zhao et al. 2013). Although ROS are crucial for eliminating bacteria from the lungs, excessive ROS production damages living cells and causes oxidative stress, which can lead to internal disorders (Valavanidis et al. 2013). In addition to particulate matter, harmful bacteria can also trigger inflammation and ROS production in the respiratory system. *Staphylococcus aureus, Pseudomonas aeruginosa*, and *Klebsiella pneumoniae* are major causes of lung infection and are implicated in the pulmonary pathology of cystic fibrosis (Riquelme and Prince 2021). PM2.5 exposure induces oxidative stress and inflammation, contributing to respiratory and systemic health issues. The use of plant‐based natural materials is important because they offer a sustainable and biocompatible approach to mitigating PM2.5‐ and bacteria‐induced damage, often exhibiting fewer side effects compared to synthetic compounds. Thus, identifying such materials with protective properties is essential for developing effective therapeutic strategies.

In East Asia, the traditional herbal medicine *Perilla frutescens* (L.) has been widely used to treat various ailments, including colds, coughs, and asthma, which are often associated with respiratory distress. This versatility is further enhanced by the genetic diversity of *Perilla frutescens* , which contributes to the variation in its bioactive compounds and therapeutic potential. Additionally, *P. frutescens* has been shown to exert substantial respiratory protective effects in various respiratory conditions, with numerous studies highlighting its potential to reduce airway inflammation and oxidative stress (Yuan et al. 2021). Perilla leaf extract (PLE) was shown to exhibit anti‐inflammatory activity by reducing inflammatory cell infiltration, cytokine levels, and ROS production (Yang et al. 2021). Additionally, perilla reportedly possesses antiviral properties and may prevent the spread of severe acute respiratory syndrome coronavirus 2 (SARS‐CoV‐2), which causes acute respiratory syndrome (Tang et al. 2021). Furthermore, compounds such as rosmarinic acid (RA) derived from perilla can target β2‐adrenergic receptors and exert antiasthmatic effects by inhibiting the NF‐κB signaling pathway (Wang et al. 2022). Despite evidence supporting the effectiveness of perilla in protecting the respiratory tract and reducing inflammation, studies on the respiratory protective effects of perilla extracts in PM_2.5_‐exposed human nasal cell models remain inadequate.

Exploring genetic resource (GR) diversity in different crops has revealed a rich spectrum of metabolites with notable antioxidant, anti‐inflammatory, and anticancer properties, highlighting the potential to improve human health through agricultural and breeding strategies. Various crops have shown a link between genetic variation and bioactive potential, emphasizing the importance of wild species as GRs for breeding programs (Raza, Hameed, and Saleem 2022). The GR diversity of perilla leaves, specifically *P. frutescens* , has been extensively studied in various regions, highlighting its importance as both an oil and vegetable crop in South Korea and other parts of East Asia (Fu et al. 2022). Despite the genetic diversity of secondary metabolites, including phenolic compounds, the development of varieties, specifically targeting their composition and functionality, remains unexplored. In Korea, 15 varieties of *P. frutescens* (commonly known as Perilla) have been developed for leaf cultivation, although these varieties have mostly been bred for their superior cultivation characteristics. Therefore, for targeted breeding of perilla leaves, it is crucial to identify useful resources from those with phenotypic diversity, such as plant traits, leaf color, aroma, secondary metabolites, and physiological activities. Although the diverse properties of various GRs of perilla leaves, encompassing antioxidant, anti‐inflammatory, antiviral, and immunomodulatory effects, indicate their potential in treating respiratory disorders, there are no studies on selecting useful perilla leaf resources through mass screening for different cultivars (cvs), elite lines (ELs), and GRs with phenotypic diversity. Therefore, the objective of this study was to (a) select a useful resource of perilla leaves ( *P. frutescens* L.) by utilizing a mass screening system using three important biomarkers (cell viability and concentrations of nitric oxide [NO] and mucin 5 AC [MUC5AC]) to identify the resource with beneficial effects against PM_2.5_‐induced respiratory disease among the 56 perilla leaf types; (b) to evaluate the respiratory protective, anti‐inflammatory, and mucus hypersecretion and fibrosis inhibitory effects of the selected resource in PM_2.5_‐induced RPMI 2650 cells. These findings may contribute to the development of functional food ingredients and breeding materials to prevent respiratory disease caused by PM_2.5_.

## 2 Materials and Methods

### 2.1 Materials

**FIGURE 1:**
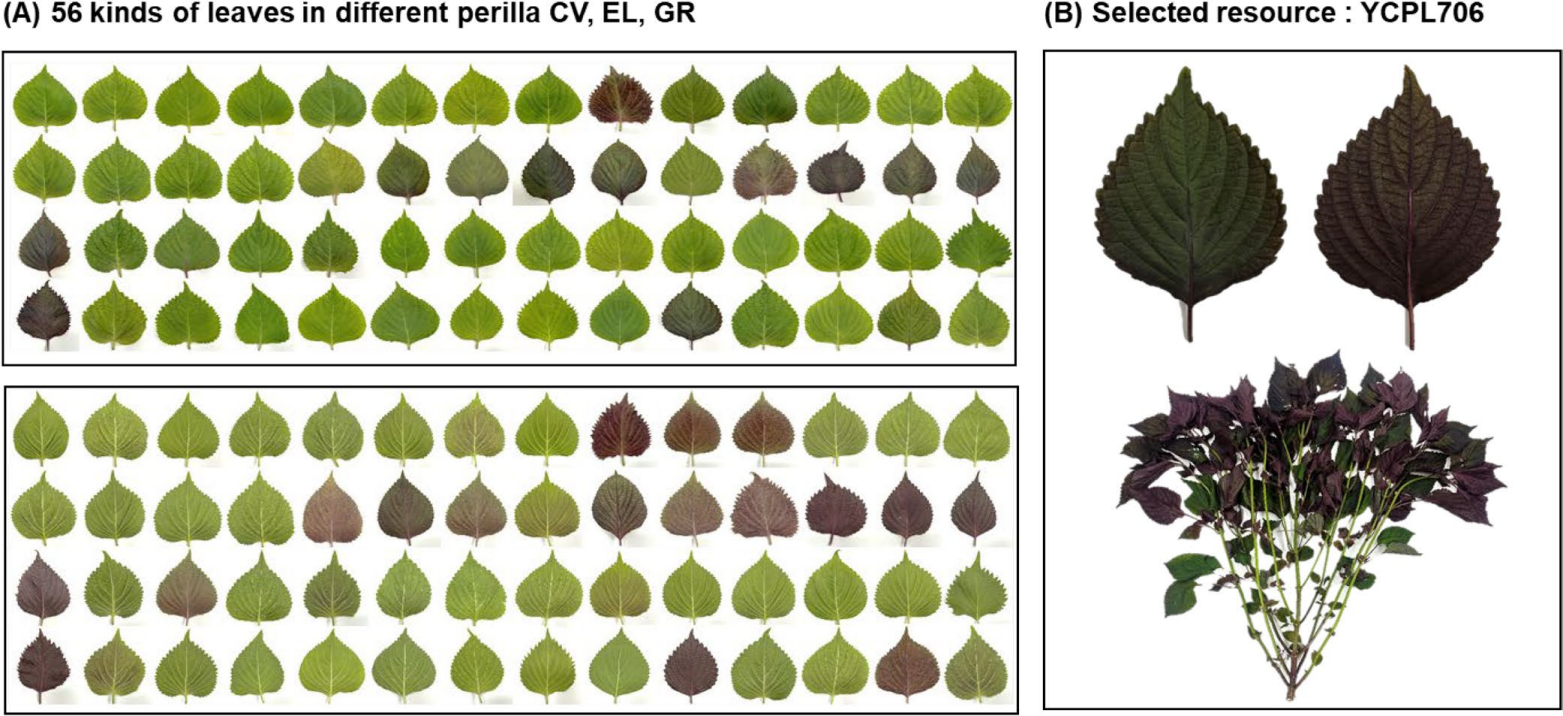
Fifty‐six leaf varieties in different cultivars (cvs), elite lines (ELs), and genetic resources (GRs) of perilla (*Perilla frutescens L*.) for mass screening of potential respiratory improvement effects.

In this study, 56 kinds of perilla leaves (categorized by cvs, ELs, and GRs) were grown at the National Institute of Crop Science in Miryang, South Korea, during the 2022 growing season. Only the leaf parts of the plants were used, which were harvested, freeze‐dried, and stored at −20°C until further analysis. An example image and list of varieties are shown in Figure 1 and Table S1, respectively. We used 11 Korean perilla cvs (cv. Yipddeulkkae1ho, cv. Namcheon, cv. Donggeun 1ho, cv. Donggeun 2ho, cv. Soim, cv. Sangyeup, cv. Somirang, cv. Saebom, cv. Neulbora, cv. Saebora, and cv. Bora), and seven ELs, and 38 GRs for a mass screening analysis of respiratory protective effects, inhibition of inflammation and mucus hypersecretion induced by PM_2.5_, such as extractable ions (Sigma, MA, USA). Three GRs (IT286242, YCPL706, and IT242103) and Namcheon were used to identify the respiratory protective effects against PM_2.5_ and antimicrobial activity against pathogens ( *S. aureus* , *P. aeruginosa* , and *K. pneumoniae* ), as recommended by the National Science Foundation in the United States. The cell line RPMI2650 was purchased from Korean Cell Line Bank (KCLB No. 10030; Seoul, Korea), while *S. aureus* (ATCC 23235), *P. aeruginosa* (ATCC 27853), and *K. pneumonia* (ATCC 13883) were purchased from American Type Culture Collection (ATCC).

### 2.2 Preparation of Flavonoid‐Rich Extracts (FREs)

FREs from 56 perilla varieties were prepared based on a previously described optimization method (Kim et al. 2023). The powdered material (2 g) was extracted twice with 40% ethanol (40 mL) at 80°C for 2 h using a shaking water bath. The extracts were then vacuum‐filtered and concentrated using a rotary evaporator, freeze‐dried, and stored in an ultra‐low freezer at −20°C.

### 2.3 Functional Compounds Analysis of Different Perilla Varieties

Total polyphenol levels were measured using the Folin–Ciocalteu (FC) method. Total polyphenol and flavonoid levels were determined according to the method described by Woo, Ko, and Jeong (2014). Briefly, 100 μL of standards or extracts were combined with 200 μL of a 2% w/v Na_2_CO₃ solution and 10 μL of a 50% v/v FC reagent (F9252; Sigma‐Aldrich, St. Louis, MO, USA). The mixtures were incubated for 30 min at room temperature, and the absorbance was recorded at 750 nm using a spectrophotometer (Multiskan GO Microplate Spectrophotometer, Thermo Fisher Scientific, Waltham, MA, USA). The results are reported in milligrams (GAE/g ER) of extract residue, which corresponds to gallic acid (G7384, Sigma‐Aldrich). Standards or extracts (50 μL) were combined with 200 μL of water and 15 μL of NaNO_2_ (5%, w/v) to evaluate total flavonoid levels. After 5 min of incubation, 30 μL AlCl_3_·6H_2_O (10% w/v) was added to the mixture and incubated for another 6 min. Subsequently, 100 μL of 1 M NaOH was added to terminate the reactions, and the absorbance at 510 nm was recorded using a spectrophotometer (Thermo Fisher Scientific). The results are expressed in milligrams of gallic acid and catechin equivalents per gram of perilla (mg GAE/g perilla leaf and mg CE/g perilla leaf). For each cultivar, individual phenolic components (luteolin [LT; 72,511], caffeic acid [CA; C0625], and RA [PHR3235]; all from Sigma‐Aldrich) were quantified using high‐performance liquid chromatography (HPLC) (UltiMate 3000 HPLC; Thermo Fisher Scientific, Waltham, MA, USA) according to the method described by An et al. (2023).

### 2.4 In Vitro Antioxidant Activities of Perilla Varieties

The radical scavenging activities of 1,1‐diphenyl‐2‐picrylhydrazyl (DPPH) and 2,2‐azinobis (3‐ethyl benzothiazoline)‐6‐sulfonic acid (ABTS) were measured using the technique described by Woo, Ko, and Jeong (2014). An aliquot (1000 μL) of 0.2 mM DPPH (549,495; Sigma‐Aldrich) methanolic solution was mixed with 50 μL of each sample, shaken vigorously, and allowed to stand in dim light for 30 min. The absorbance was then measured at 515 nm. Cationic ABTS radicals were generated by adding 7 mM ABTS (A1888; Sigma‐Aldrich) to 2.45 mM potassium persulfate solution (216,224; Sigma‐Aldrich) and storing the mixture overnight in the dark at room temperature. The radical solution was diluted with methanol to achieve an absorbance of 1.4–1.5 at 735 nm (molar extinction coefficient, ε = 3.6 × 104/mol/cm). The diluted ABTS radical solution (1000 μL) was added to 50 μL of each extract, Trolox standard solution, or distilled water. After 30 min, absorbance was measured spectrophotometrically at 735 nm (Multiskan GO Microplate Spectrophotometer; Thermo Fisher Scientific). Both radical scavenging activities are expressed as Trolox‐equivalent antioxidant capacity (TEAC) in mg TE/g sesame (ER). Upon treatment like the Trolox mixture, the antioxidant capacities of the sample extracts were calculated as the Trolox equivalent antioxidant capacity (TEAC) using a calibration curve: *y* = −0.0043*x* + 1.1268, R2 = 0.9990 (DPPH), *y* = −0.0058*x* +0.9999, R2 = 0.9974 (ABTS).

### 2.5 Mass Screening Analysis for Respiratory Disease Enhancement Effect

The cell line RPMI2650, derived from squamous cell carcinoma of the nasal septum, was purchased from the Korean Cell Line Bank (KCLB No. 10030; Seoul, Korea). RPMI2650 cells were grown in Roswell Park Memorial Institute (RPMI) 1640 supplemented with 10% fetal bovine serum (FBS), 100 U/mL penicillin, and 50 μg/mL streptomycin in an incubator containing 5% CO_2_ atmosphere at 37°C. The cells were subcultured in 0.05% trypsin‐ethylenediaminetetraacetic acid (EDTA) and phosphate‐buffered saline (PBS). The cytotoxicity of PM_2.5_ (25, 50, 100, 200, and 400 μg/mL) and FREs (25, 50, 100, 200, and 400 μg/mL) from perilla leaves of Namcheon, and FREs (100 μg/mL) from 56 perilla leaf varieties were analyzed in RPMI2650 cells using the 3‐[4,5‐dimethylthiazol‐2‐yl]‐2,5 diphenyl tetrazolium bromide (MTT) assay (Ishiyama et al. 1996; Xing et al. 2016). After identifying the optimal PM_2.5_ (100 μg/mL) and FREs (100 μg/mL) from the perilla leaves of Namcheon, we evaluated the PM_2.5_‐induced respiratory protective effects using a mass screening system. Cytoprotective effect (cell viability, %), inflammatory response (NO concentration, μM), and mucus production (MUC5AC, ng/mL) were determined. RPMI2650 cells were cultured in a 96‐well plate (5 × 10^4^ cells/well) to determine their cytoprotective effects against PM2.5 (100 μg/mL), with experiments performed in triplicate to ensure reliability and reproducibility. After 24 h, Cells were treated with the positive control, i.e., thymoquinone (03416; Sigma‐Aldrich), which was selected for its well‐documented anti‐inflammatory effects in respiratory systems (Darakhshan et al. 2015), along with FREs (100 μg/mL) from 56 perilla leaf varieties and PM2.5 (100 μg/mL). After 24 h incubation, cell viability was determined using the MTT assay. Simultaneously, the concentrations of NO and MUC5AC in the cell culture supernatants were measured using a Griess Reagent System (Promega, Madison, WI, USA) and an ELISA kit (Abcam, Cambridge, UK), respectively.

### 2.6 PM_2_ _.5_‐Induced Respiratory Protective Effects of Selected Resources in RPMI2650 Cells

Human nasal RPMI2650 cells were cultured in a 96‐well plate (2 × 10^4^ cells/well) to determine the cytoprotective effect against PM_2.5_ (100 μg/mL) (Hong et al. 2016). After 24 h, the cells were treated with PLE (50 and 100 μg/mL) from three perilla resources and Namcheon, along with suspended PM_2.5_ (100 μg/mL). After 24 h of incubation, cell viability was determined using the MTT assay as described previously. RPMI2650 cells cultured in a 24‐well plate (1 × 10^6^/well) were used for detecting intracellular ROS levels, lipid peroxidation, and antioxidant activity. Intracellular ROS levels were quantified using a 2′,7′‐dichlorodihydrofluorescein diacetate (DCFH‐DA) fluorescent probe as described previously (Jin et al. 2018). The fluorescence intensity corresponding to intracellular ROS generation was measured using a fluorescence spectrophotometer for 2 h at excitation and emission wavelengths of 485 and 530 nm, respectively. Cells were harvested and lysed using radioimmunoprecipitation assay buffer (RIPA; Sigma, MA, USA) to determine lipid peroxidation and antioxidant activity. Lysates were centrifuged (10,000 × *g*, 10 min, 4°C), and supernatants were used for protein and lipid peroxidation and antioxidant assays (Crobeddu et al. 2020).

### 2.7 PM_2_ _.5_‐Induced Anti‐Inflammatory Effects in RPMI2650 Cells From Selected Resources

Human nasal RPMI2650 cells cultured in a 24‐well plate (1 × 10^6^/well) were used to analyze NO and cytokine (tumor necrosis factor‐alpha [TNF‐α], interleukin [IL]‐6, and IL‐1β) production (Lyu et al. 2022). The cells were treated with suspended PM_2.5_ (100 μg/mL) and PLEs (50 and 100 μg/mL) from three perilla resources (IT286242, YCPL706, and IT242103) and Namcheon after a 24‐h period. Following a 24‐h incubation period, the concentrations of NO and cytokines in cell‐free supernatants were determined using a Griess Reagent System (Promega, Madison, WI, USA) and an ELISA kit, respectively (R&D Systems, Abingdon, UK), following manufacturer's instructions.

### 2.8 PM_2_ _.5_‐Induced Inhibitory Effects of Mucus Hypersecretion and Fibrosis in RPMI2650 Cells of Selected Resources

Human nasal RPMI2650 cells cultured in a 12‐well plate (2 × 10^6^/well) were used to evaluate mucus hypersecretion and fibrosis inhibition induced by PM_2.5_ (Song et al. 2015). Mucus hypersecretion and fibrosis were assessed by analyzing intracellular MUC5AC and matrix metalloproteinase (MMP)‐9 (MMP‐9) expression, respectively. The cells were treated with suspended PM_2.5_ (100 μg/mL) and PLEs (50 and 100 μg/mL) from three perilla resources (IT286242, YCPL706, and IT242103) and Namcheon after a 24‐h period. Following a 24‐h incubation period, the cells were harvested and lysed using RIPA (Sigma, MA, USA) to determine MUC5AC and MMP‐9 concentrations. Lysates were centrifuged (10,000 × *g*, 10 min, 4°C), and supernatants were analyzed to determine MUC5AC and MMP‐9 concentrations using an ELISA kit (R&D Systems, Abingdon, UK) following the manufacturer's instructions.

### 2.9 Antimicrobial Activities of Selected Resources

Antimicrobial susceptibility testing of PLEs from three sources (IT286242, YCPL706, and IT242103) and Namcheon against *S. aureus* (ATCC 23235), *P. aeruginosa* (ATCC 27853), and *K. pneumoniae* (ATCC 13883) was performed using the standard broth microdilution method (Nguyen et al. 2020). The minimum inhibitory concentration (MIC) was the lowest drug concentration that inhibited visible growth. Additionally, the antibacterial activities were assessed using the disk diffusion method. Bacterial cultures were diluted to 0.5 McFarland standard with 0.9% NaCl. A bacterial suspension was swabbed onto Mueller‐Hinton broth using a sterile cotton bud. The culture was incubated at 37°C for 24 h. The inhibition zone diameter was measured using a caliper. A paper disk containing various essential oils prepared from natural banana peel paper was placed on a Petri dish containing the bacterial medium. The Petri dish was incubated at 30°C for 24 h. The zone of inhibition was defined as the distance from the free area around the disk.

### 2.10 Statistical Analysis

All data are expressed as mean ± standard deviation (SD). Significant differences between treatments were determined using one‐way analysis of variance (ANOVA) with Duncan's multiple range test by employing SAS ver. 9.2 software (SAS Institute, Cary, NC, USA). The significance level was set at 0.05. For correlation analysis and principal component analysis (PCA), loading plots, score plots, and heatmaps were generated to assess the results using the MetaboAnalyst 5.0 platform for metabolomics data analysis (single factor; https://www.metaboanalyst.ca/home.xhtml).

## 3 Results and Discussion

### 3.1 Screening of Functional Compounds and Antioxidant Activity of Perilla Leaf Varieties

**FIGURE 2:**
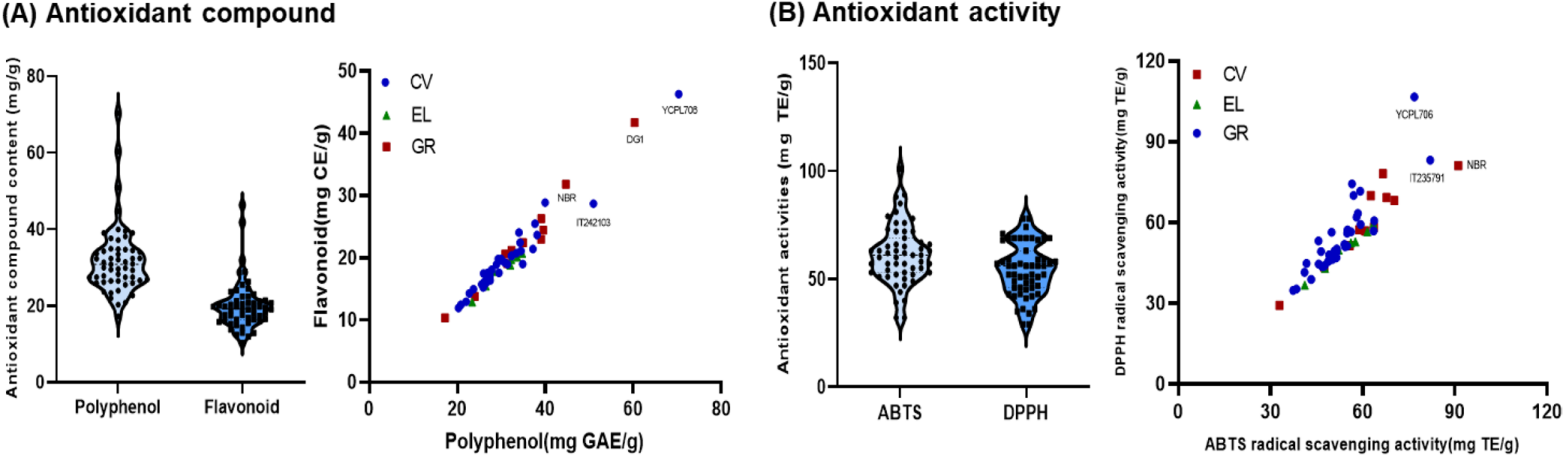
Distribution of (A) antioxidant compounds (polyphenols and flavonoids) and (B) antioxidant activities (2,2‐azinobis (3‐ethyl benzothiazoline)‐6‐sulfonic acid [ABTS] and 1,1‐diphenyl‐2‐picrylhydrazyl [DPPH] radical scavenging activity, mg TE/g) in 56 perilla leaf varieties (*Perilla frutescens L*.) from cultivars (cvs), elite lines (Els), and genetic resources (GRs).

**FIGURE 3:**
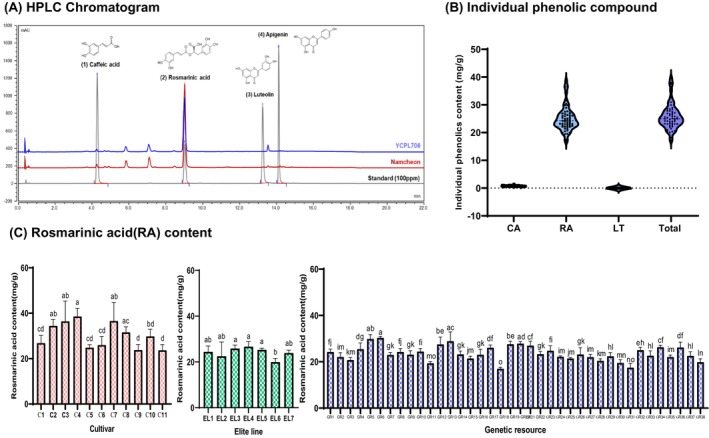
Distribution of individual phenolic compounds (caffeic acid [CA], rosmarinic acid [RA], luteolin [LT]) in 56 perilla leaf varieties (*Perilla frutescens L*.) from cultivars (cvs), elite lines (Els), and genetic resources (GRs).

Perilla leaves are traditionally used for their antioxidant, anti‐glycosuric, antiallergic, antimicrobial, and anticancer properties, which are attributed to the presence of phenolic compounds, such as RA, anthocyanins, essential oils, vitamins, and minerals (Bao Thy et al. 2022). Therefore, prior to assessing the potential respiratory protective effects, we detected the amounts of polyphenols, flavonoids, and specific phenolics, including CA, RA, and LT, as well as their bioactive contributions in 56 different perilla leaf varieties. Figure 2 and Table S1 illustrate the distribution of total polyphenolic content (TPC), total flavonoid content (TFC), ABTS, and DPPH radical scavenging activity. The TPC and TFC of the perilla leaf varieties were 17.22–70.29 mg GAE/g and 10.37–46.30 mg CE/g, respectively. YCPL706 and Korean cv Donggeun 1ho exhibited the highest TPC (70.29 and 60.27 mg GAE/g) and TFC (46.30 and 41.77 mg CE/g) values. The ABTS and DPPH radical scavenging activity of the perilla leaf varieties were 32.28–101.31 and 29.20–78.13 mg TE/g, respectively. YCPL706 and IT286242 of GR had the highest ABTS (101.32 and 89.02 mg TE/g) and DPPH radical scavenging activities (78.13 and 41.77 mg CE/g), respectively. The coefficient values (CV) of TPC, TFC, ABTS, and DPPH radical scavenging activities in the perilla varieties were 28.39, 31.77, 22.17, and 22.19, respectively. This confirmed the diversity of antioxidant components and antioxidant activities. In addition, individual phenolic compounds of perilla leaf cultivars were evaluated using HPLC (Figure 3A and Table S2). The results showed that the standard contained three phenolic compounds (CA, RA, and LT), with RA comprising the highest percentage. The RA concentrations in cvs, ELs, and GRs were 23.72–38.65 mg/g, 19.99–26.71 mg/g, and 17.00–28.94 mg/g, respectively (Figure 3B). Donggeun 1ho had the highest RA concentration (38.65 mg/g), followed by Somirang (36.61 mg/g), Donggeun 2ho (36.61 mg/g), and Namcheon (34.50 mg/g) of Korean cv (Figure 3C). According to An et al. (2023), TPC and RA in 90 perilla leaf varieties from Korea were 13.19–35.85 mg GAE/g and 6.20–39.47 mg/g, respectively. The antioxidant properties of perilla are predominantly attributed to its enrichment in flavonoids, phenolic compounds, and other polyphenolic chemicals, which vary depending on the cultivator and growing environment. Studies have identified major metabolites, such as palmitic acid, stearic acid, linoleic acid, linolenic acid, rosmarinyl glucoside, and RA, notably contribute to the antioxidant capacity of perilla leaves (Gu et al. 2019). Therefore, these variations in the antioxidant properties of perilla leaf varieties could influence physiological properties, including respiratory protective effects against PM_2.5_‐induced damage in RPMI2650 cells.

### 3.2 Screening of Respiratory Improvement Effects in Perilla Leaf Varieties

The inflammatory and pathological biomarkers secreted by RPMI2650 cells were analyzed in response to different PM_2.5_ concentrations to establish a mass screening system and analyze respiratory protection effects. With increasing PM_2.5_ concentrations (25–400 μg/mL), the viability of the nasal epithelial cell line and the secretion of inflammatory cytokines (TNF‐α, IL‐6, and IL‐1β), mucus‐related biomarkers (MUC5AC) decreased, whereas levels of ROS and fibrosis‐related biomarkers (MMP‐9) increased (data not shown). Specifically, stimulation of RPMI2650 cells with PM_2.5_ (100 μg/mL) reduced cell viability to approximately 50% and significantly increased biomarker secretion related to respiratory health. This established a cellular model for a mass screening system to analyze the aggravating effects of respiratory disease. The barrier formed by the sinonasal and nasal epithelial cells is a vital defense mechanism against air pollution particles. Elevated PM_2.5_ levels can cause oxidative stress, alter mitochondrial metabolism, and damage intercellular connections, all of which could lead to disruption of the nasal epithelium (Zaręba et al. 2024). Nasal epithelial cells undergo inflammatory changes following exposure to PM_2.5_ (Xian et al. 2020; Ramanathan Jr et al. 2017).

**FIGURE 4:**
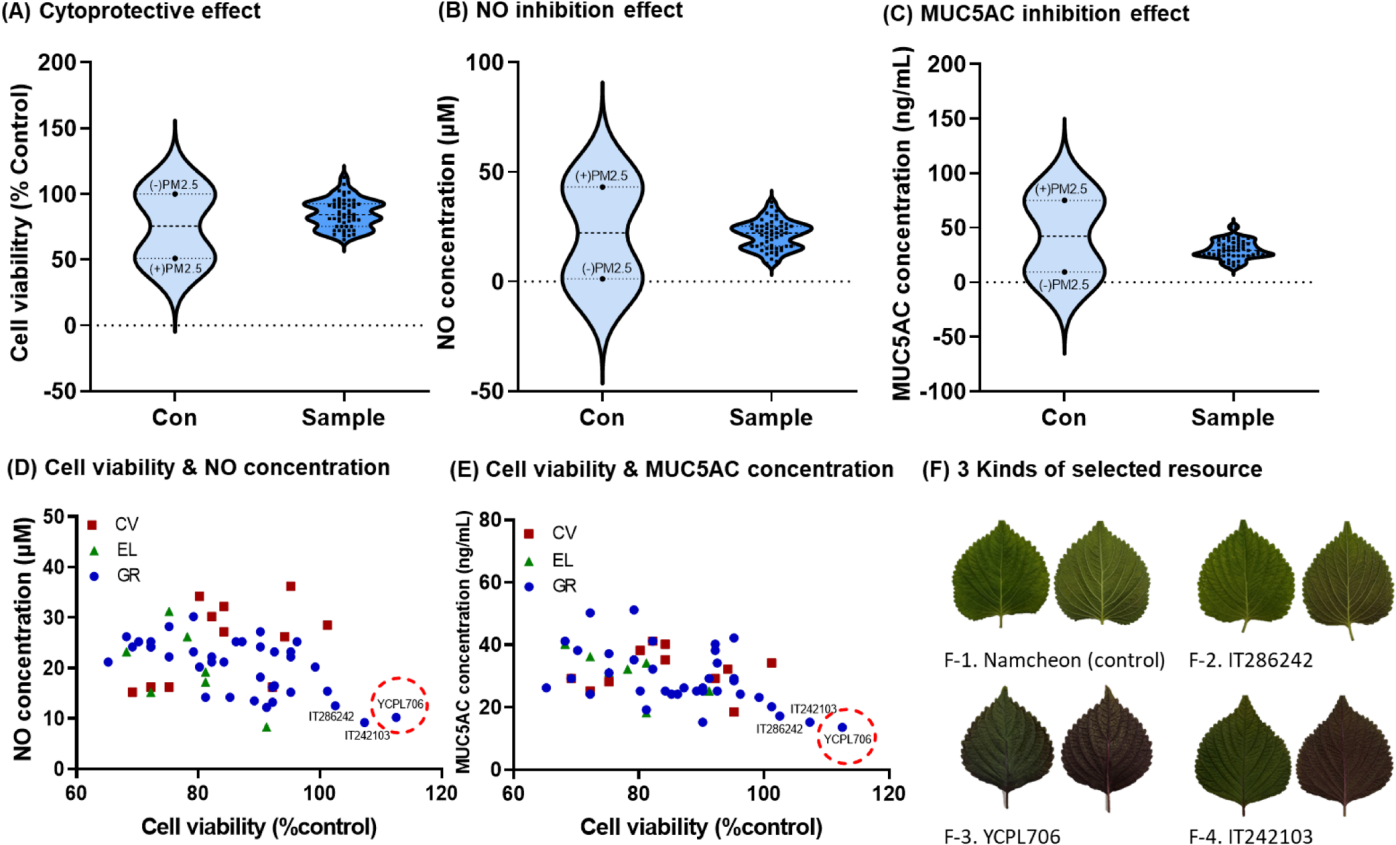
Selection of useful resources for perilla leaves (*Perilla frutescens L*.) through a mass screening system for beneficial effects against fine particulate matter (PM_2.5_)‐induced respiratory disease. (A) Cell viability, (B) nitric oxide (NO), and (C) secretion of mucin 5 AC (MUC5AC) in human nasal cells (RPMI2650) exposed to PM_2.5_. (D) Cell viability & NO concentration, (E) cell viability & MUC5AC concentration, and (F) three selected kinds of resources.

To determine the respiratory health benefits of the positive control (thymoquinone, 2.5 ppm) and Namcheon, we examined the effects on respiratory protection, inhibition of inflammatory cytokine secretion, and reduction of mucin production against PM_2.5_‐exposed RPMI2650 cells. PLEs did not induce cytotoxicity at evaluated at examined concentrations (≤ 200 μg/mL). In RPMI2650 cells, PM_2.5_ exposure reduced cell viability by 51% and increased the secretion of TNF‐α, IL‐6, MUC5AC, key biomarkers of inflammation, and mucus hypersecretion. However, treatment of cells exposed to PM_2.5_ (100 μg/mL) with Namcheon PLE significantly enhanced cell viability while reducing inflammatory cytokine and mucin secretion in a concentration‐dependent manner, except at 200 μg/mL. This confirmed the effectiveness of extracts in protecting the respiratory tract from the negative effects of PM_2.5_ exposure, which promotes inflammation and mucus hypersecretion. Thymoquinone, which is present in *Nigella sativa* and used as a positive control in this work, reportedly reduces inflammation, oxidative stress, apoptosis, and autophagy in PM_2.5_‐induced lung injury by decreasing inflammatory response markers (IL‐1β, IL‐6, and TNF‐α), and oxidative stress (Mao et al. 2020). Additionally, the antiallergic and anti‐inflammatory effects of compounds derived from *P. frutescens* have been reported in numerous in vitro and in vivo models (Yu et al. 2017). Specifically, PLEs were found to exert substantial anti‐inflammatory effects in non‐tumorigenic human bronchial epithelial cells (BEAS‐2B) (Liu et al. 2013). In *Dermatophagoides pteronyssinus* 2 (DP2)‐stimulated BEAS‐2B cells, treatment with PLEs dramatically reduced the mRNA expression and protein levels of pro‐allergic and pro‐inflammatory cytokines by blocking P38/c‐Jun N‐terminal kinases (JNK) and NF‐kB activation. Although perilla leaf‐derived compounds are useful as functional materials for suppressing inflammation and respiratory disease in the nasal passages, bronchi, and lungs, studies exploring the respiratory protective effects of perilla extracts in PM_2.5_‐exposed human nasal cell models remain inadequate. Additionally, there have been no studies on selecting useful perilla leaf resources through mass screening for various cvs, ELs, and GR with phenotypic diversity, such as plant traits, leaf color, aroma, and secondary metabolites. The genetic diversity of the collected perilla leaves could lead to the accumulation of various secondary metabolites, which may afford a wide range of health benefits. Therefore, the three biomarkers (cell viability, NO, and MUC5AC concentration) were evaluated using a mass screening system to identify potential respiratory protective effects of 56 perilla leaf varieties produced in 2022, including cvs, ELs, and GR (Figure 4), in PM_2.5_‐induced RPMI2650 cells. Preliminary experiments were performed to determine the toxic levels of PM_2.5_ and PLEs in RPMI2650 cells using the MTT assay (Table S1). Although exposure of RPMI2650 cells to PM_2.5_ (100 μg/mL) resulted in 46.48% cytotoxicity when compared with control cells, no cytotoxicity was observed upon treatment of cells with < 200 μg/mL of PLEs of all varieties. Therefore, PM_2.5_ at 100 μg/mL was selected to induce cell death in subsequent experiments. Next, we analyzed the cytoprotective and NO and MUC5AC inhibitory effects of PLEs of 56 varieties, including cvs, ELs, and GR, against PM_2.5_‐induced toxic damage, and these effects differed between the 56 varieties (Figure 4A, Table S3). Cell viability was reduced to 46.5% of normal cells upon PM_2.5_ stimulation but increased to 65.2%–112.5% upon treatment with PLEs of 56 varieties. The concentration of NO, a notable inflammatory marker secreted by nasal cells, increased from 6.74 to 41.03 μM upon PM_2.5_ exposure but decreased to 8.3–36.2 μM in PLEs from 56 different varieties. The concentration of MUC5AC, a crucial marker of mucus hypersecretion by nasal cells, increased from 10.28 to 64.63 ng/mL upon PM_2.5_ exposure but decreased to 13.6–51.2 ng/mL upon PLE treatment. Among the 56 extracts, three GRs (IT286242, YCPL706, and IT242103) showed significant respiratory protective effects against fine dust stimulation while effectively inhibiting levels of NO (an indicator of severe inflammation; Figure 4D) and MU5AC (an indicator of mucus hypersecretion; Figure 4E). When examining the properties of the highly active GRs, IT286242 exhibited a high polyphenol content; YCPL706 was a scarce resource with notable aromatic properties and a unique purple color; and IT242103 had wrinkled leaves with a high flavone content (e.g., LT). Among 56 leaf varieties of *P. frutescens* , *P. frutescens Britton* var. *acuta Kudo*, which has purple leaves and a distinctive aroma, was selected. It is called red perilla, a traditional East Asian medicinal herb, and shows therapeutic efficacy against various complex disorders, including allergies, cancer, pathogenic bacteria, asthma, obesity, dyslipidemia, and systemic damage caused by oxidative stress (Makino et al. 2003). Epidemiological studies on *P. frutescens Britton var. acuta Kudo* have confirmed its effectiveness against various diseases (He, Yao, and Chang 2015). The distinctive color and aroma of *P. frutescens* Britton *var. acuta Kudo* can be attributed to the expression of various biochemical compounds (Makino et al. 2003), and the expression of these components may play a notable role in potential health benefits exerted by this plant. The plant color, especially the presence of red or purple hues in its leaves, is primarily due to the presence of anthocyanins, a group of pigments with antioxidant properties. The aroma of *P. frutescens* *var. acuta* is driven by the presence of volatile organic compounds, such as methyl benzoate and limonene, which are often linked to its aromatic profile. However, it cannot be conclusively established that the respiratory health activity of the YCPL706 resource is solely due to the presence of anthocyanins and aromatic compounds. Further studies are needed to elucidate the underlying mechanism by employing bioassay‐based isolation. Through mass screening experiments, we identified three GRs in *P. frutescens Britton var. acuta Kudo* that exert distinct effects capable of improving respiratory health. We examined their respiratory protective and anti‐inflammatory effects, as well as their capacity to inhibit mucus hypersecretion and fibrosis, in PM_2.5_‐induced RPMI2650 cells.

### 3.3 Correlation Analysis and PCA

**FIGURE 5:**
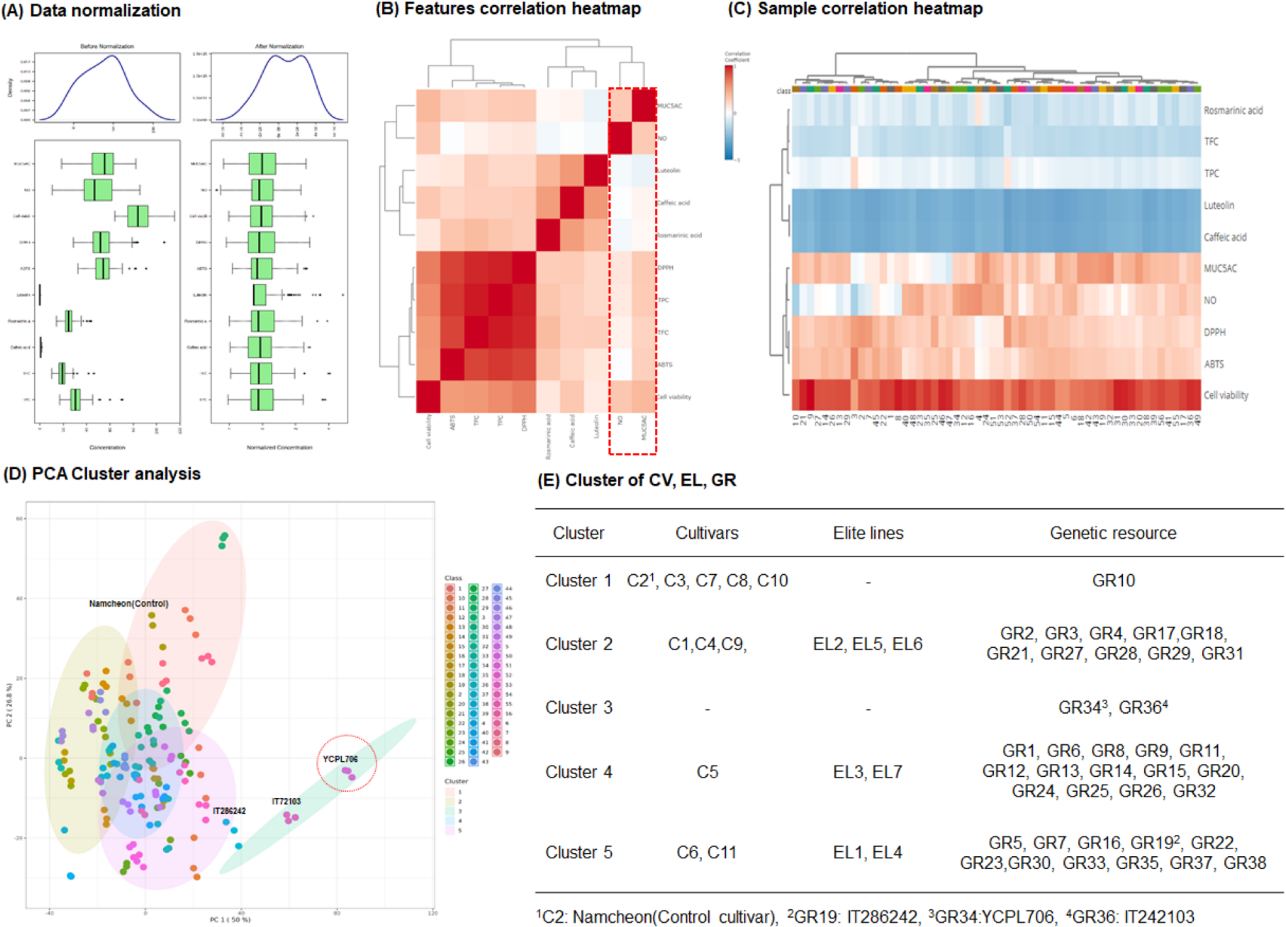
Correlation heat map analysis and principal component analysis (PCA) of the associated antioxidant compounds and activities, individual phenolic compounds, and protective effects against respiratory disease obtained from 56 leaf varieties (*Perilla frutescens L*.) in different cultivars (cvs), elite lines (Els), and genetic resources (GRs).

We performed correlation analysis and PCA using Metaboanlayst 5.0 for the results of TPC, TFC, individual phenolics (RA, CA, and LT), antioxidant activities (ABTS and DPPH radical scavenging activity), cell viability, NO, and MUC5AC inhibitory activity of 56 perilla leaf varieties. Figure 5B presents a heatmap illustrating the correlation between functional compounds and significant factors affecting the antioxidant and respiratory protective properties of PLEs of different varieties. Comprehensive compounds and antioxidant activities of perilla leaves, including TPC, TFC, ABTS, and DPPH radical scavenging activities, showed significant positive correlations with cell viability, NO, and MUC5AC inhibition. However, individual phenolic compounds, such as RA, CA, and LT, exhibited conflicting correlation patterns with significant factors influencing respiratory function improvement. Among individual phenolic compounds, RA, a major phenolic acid component of *P. frutescens* , exhibits a wide range of pharmacological activities, including antimicrobial, antioxidant, anti‐inflammatory, and potential therapeutic effects against various diseases (Xu et al. 2023). However, compounds other than RA interfere with the health benefits of *P. frutescens* and *P. frutescens Britton var. acuta Kudo*. For example, luteolin glucosides, specifically luteolin‐7‐O‐glucoside and its derivatives, are potent compounds with substantial therapeutic potential for eye health (Yang et al. 2020). Perilla‐derived methoxyflavone reportedly induces p53‐mediated G2/M cell cycle arrest and apoptosis in A549 lung adenocarcinoma, indicating its potential as an anticancer compound (Traina et al. 2022). Based on these correlation analysis results, we hypothesized that the respiratory health benefits evaluated in this study may be due to compounds other than the main compounds (i.e., RA, CA, and LT). Further studies are underway to isolate and identify compounds using relevant bioassays. PCA is commonly used to synthetically analyze clustering trends in multidimensional data. PCA was employed to evaluate the clustering and variations of functional compounds and key factors affecting the antioxidant and respiratory protective properties of 56 perilla leaf varieties (Figure 5D). PC1 and PC2 accounted for 50% and 26.8% of the variance, respectively. Cluster analysis showed that the three selected GRs (IT286242, YCPL706, and IT242103) were divided into different clusters. By performing mass screening experiments and cluster analysis, we investigated the respiratory protective, anti‐inflammatory, and inhibitory effects on mucus hypersecretion and fibrosis of selected compounds in PM_2.5_‐induced RPMI2650 cells.

### 3.4 Respiratory Protective Effects of Selected Resources in PM_2_ _.5_‐Induced RPMI2650 Cells

**FIGURE 6:**
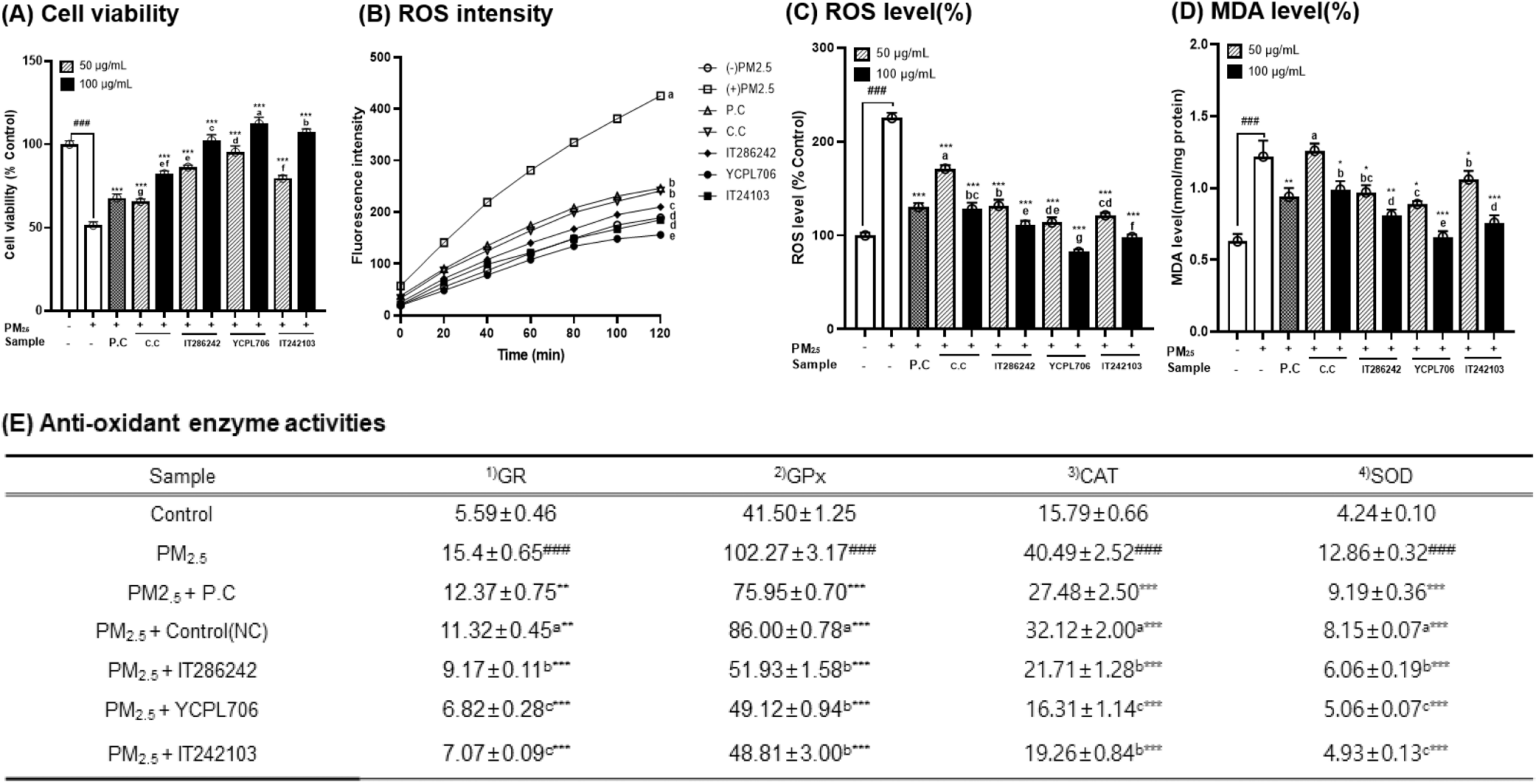
Respiratory protective effects of selected genetic resources (I286242, YCPL706, and IT242103) in fine particulate matter (PM_2.5_)‐induced RPMI2650 cells compared to the control cultivar (Namcheon) and positive control (P.C; thymoquinone 2.5 ppm). Assessment of (A) cell viability, (B) reactive oxygen species (ROS) fluorescence intensity, (C) intracellular ROS levels, (D) lipid peroxidation of malonaldehyde (MDA; nmol/mg protein), and (E) antioxidant enzyme activity of glutathione reductase (GR; μmol/min/mg protein), glutathione peroxidase (GPX; μmol/min/mg protein), catalase (CAT; μmol/min/mg protein), and superoxide dismutase (SOD; unit/mg protein) in RPMI2650 cells treated for 24 h with the sample and 100 μg/mL PM_2.5_. Values are the mean ± SD of 3 replicates. Different small letters in the same items indicate a significant difference (*p* < 0.05) among different perilla resources and concentrations of extracts. ^*###*^ *p* < 0.001 and ***p < 0.001 represents significant difference compared to PM2.5 treated control.

Direct exposure to PM_2.5_ causes oxidative stress in keratinocytes, bronchial, and alveolar epithelial cells by inducing ROS generation and lipid peroxidation (Traina et al. 2022). The respiratory protective effects of PLEs from selected GRs (IT286242, YCPL706, and IT242103) and Namcheon on PM_2.5_‐induced damage in RPMI2650 cells were evaluated. Cell viability, ROS production, malondialdehyde (MDA) concentration, and antioxidant enzyme activity (glutathione reductase [GR], glutathione peroxidase [GPX], superoxide dismutase [SOD], and catalase [CAT]) were evaluated (Figure 6). Prior to assessing the respiratory protective effect, cytotoxicity tests were performed using the MTT assay to determine the optimal concentrations (50 and 100 μg/mL) of PLEs from the selected GRs and control cvs. Subsequently, the cytoprotective effects of PLEs against PM_2.5_‐induced oxidative damage were evaluated using four different resources. RPMI2650 cells were treated with two‐point concentrations of samples (50 and 100 μg/mL) containing PM_2.5_ (100 μg/mL) for 24 h. Herein, treatment with PM_2.5_ (100 μg/mL) significantly reduced cell viability by 51.31% compared with that of control cells. However, treatment with PLE at 50 and 100 μg/mL significantly increased cell viability in a dose‐dependent manner, regardless of the four varieties. In particular, PLEs of YCPL706 exerted the highest protective effects against PM_2.5_‐induced RPMI2650 cell damage, increasing cell viability to 112.37%. Exposure to PM_2.5_ significantly stimulated oxidative cell stress and damage and increased ROS generation in RPMI2650 cells by 225.18% when compared with that in the control cell without PM_2.5_ exposure. However, treatment with PLEs at 50 and 100 μg/mL significantly and dose‐dependently reduced PM_2.5_‐induced intracellular ROS levels in RPMI2650 cells. Specifically, at a concentration of 100 μg/mL, YCPL706 extract reduced ROS levels from 225.15% to 82.05% in Aβ‐induced cell stress. Subsequently, the PM_2.5_‐induced intracellular MDA concentration in RPMI2650 cells was analyzed as an index of lipid peroxidation. Additionally, intracellular antioxidant enzymes play a crucial role in the defense mechanisms against oxidative damage. In RPMI2650 cells, PM_2.5_ exposure‐induced oxidative stress substantially increased the MDA concentration and enhanced enzyme activities of GR, GPX, CAT, and SOD (Figure 6E); however, these increments were counteracted by pretreatment with PLEs (100 μg/mL). The PLEs of YCPL706 most effectively counteracted the increased MDA concentration and enhanced GR, GPX, CAT, and SOD activities. In the current study, PLEs, specifically selected GR (YCPL706) exhibiting respiratory protective activity distinct from common perilla leaves, effectively increased cell viability in the presence of PM_2.5_ exposure, decreased ROS levels, and suppressed lipid peroxidation and intracellular antioxidant enzymes. Yuan et al. (2021) reported that PLE reduced intracellular ROS production in respiratory cells, suggesting possible antioxidant effects in chronic obstructive pulmonary disease (COPD) by reducing leukocytes and inflammatory mediators and the involvement of the TLR4/Syk/PKC/NF‐κB p65 as a potential mechanism. Additionally, Pintha et al. (2021) found that the RA‐rich fraction derived from perilla reduced ROS production in A549 lung cells exposed to PM2.5, indicating potential respiratory benefits against PM2.5‐induced oxidative stress. Furthermore, RA and LT have been shown to inhibit pro‐inflammatory cytokine production, such as TNF‐α and IL‐6, while enhancing antioxidant enzyme activities, including SOD and CAT (Kim et al. 2019). Various studies have consistently reported the respiratory protective effects of perilla leaves in PM2.5‐induced bronchial and lung models, primarily attributed to the phenolics, such as RA and LT. To the best of our knowledge, no study has elucidated the respiratory protective effects of perilla extracts in a PM_2.5_‐exposed human nasal cell model, and there have been no studies on selecting useful perilla leaf resources. The results of this study demonstrate that YCPL706 effectively protects against oxidative damage in RPMI2650 cells by modulating ROS production, MDA generation, and antioxidant enzyme activities.

### 3.5 Inhibition of Inflammation, Mucus Hypersecretion, and Fibrosis by Selected Resources in PM_2_ _.5_‐Induced RPMI2650 Cells

**FIGURE 7:**
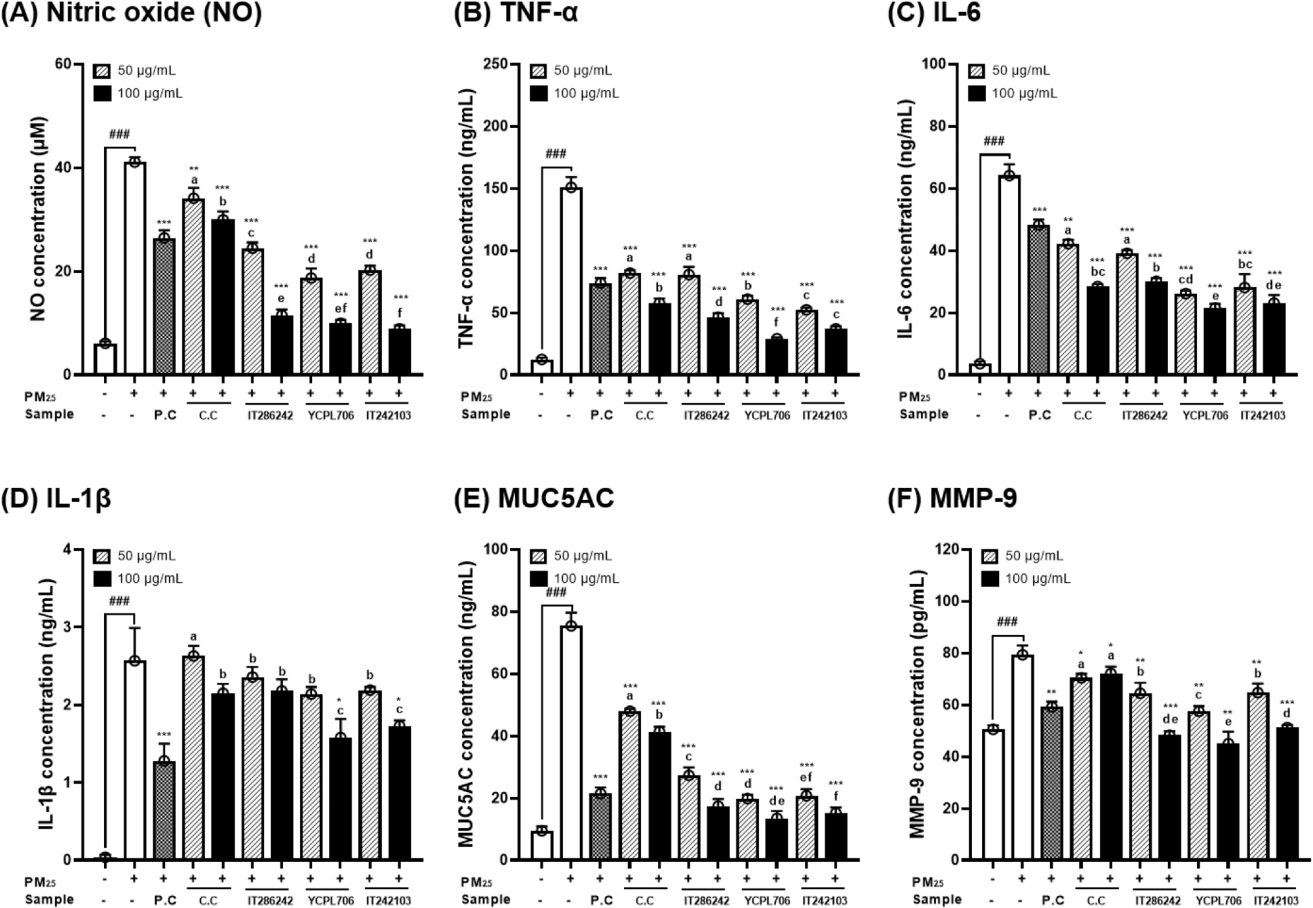
Inhibitory effects of selected genetic resources (I286242, YCPL706, and IT242103) on fine particulate matter (PM_2.5_)‐induced inflammation, mucus hypersecretion, and fibrosis in RPMI2650 cells compared with the control cultivar (Namcheon) and positive control (P.C; thymoquinone 2.5 ppm). Assessment of (A) nitric oxide (NO), (B) tumor necrosis factor‐alpha (TNF‐α), (C) interleukin (IL)‐6, (D) IL‐1β, (E) mucin 5 AC (MUC5AC), and (F) matrix metalloproteinase‐9 (MMP‐9) levels in RPMI2650 cells treated for 24 h with the sample and 100 μg/mL PM_2.5_. Values are the mean ± SD of 3 replicates. Different small letters in the same items indicate a significant difference (*p* < 0.05) among different perilla resources and concentrations of extracts. ^*###*^ *p* < 0.001 and ***p < 0.001 represents significant difference compared to PM2.5 treated control.

Chronic intranasal exposure to fine dust causes lung inflammation, mucus hypersecretion, and pulmonary fibrosis. Additionally, this exposure leads to oxidative stress, associated with the TGFβ1‐PI3K/Akt, TGFβ1‐NOX, and TGFβ1‐NLRP3 signaling pathways, affecting mucus production and fibrosis (Xu et al. 2021). Therefore, we examined the inhibitory effects of PLEs of selected GRs (IT286242, YCPL706, and IT242103) and Namcheon on inflammation, mucus hypersecretion, and fibrosis using PM_2.5_‐induced RPMI2650 cells by assessing levels of NO (Figure 7A), TNF‐α (Figure 7B), IL‐6 (Figure 7C), IL‐1β (Figure 7D), MUC5AC (Figure 7E), and MMP‐9 (Figure 7F). Nitrite is a product of NO oxidation that acts as a regulator of the inflammatory response. Excessive nitrite production reacts with superoxide, causing cytotoxicity and tissue damage (Kevil and Lefer 2010). Additionally, TNF‐α, IL‐6, and IL‐1β are crucial pro‐inflammatory cytokines associated with the pathogenesis of numerous infectious and inflammatory diseases, including cancer (Bertazza and Mocellin 2008). As illustrated in Figure 6A–D, the production of NO, TNF‐α, IL‐6, and IL‐1β was significantly upregulated in PM_2.5_‐induced RPMI2650 cells. Upon exposure to PM_2.5_, the concentration of NO, TNF‐α, IL‐6, and IL‐1β in the medium of untreated RPMI2650 cells (6.12 μM, 12.46 ng/mL, 3.60 ng/mL, and 0.04 ng/mL, respectively) was significantly increased to 41.12 μM, 150.98 ng/mL, 64.17 ng/mL, and 2.57 ng/mL, respectively. However, treatment with perilla leaf extracts significantly reduced NO, TNF‐α, IL‐6, and IL‐1β levels and significantly increased cell viability in a dose‐dependent manner, regardless of the four varieties. Specifically, perilla leaf extracts of YCPL706 effectively reduced the concentration of NO, TNF‐α, IL‐6, and IL‐1β by 9.98 μM, 29.17, 8.46, 21.50, and 1.58 ng/mL, respectively. YCPL706, characterized by its notable aroma and unique purple color, exerted an effective respiratory protective effect against PM_2.5_‐induced inflammation in RPMI2650 cells. Lim et al. (2014) reported that components of *P. frutescens*, such as elemicin and myristicin, inhibit IL‐6 production in lung cells, indicating the potential to treat respiratory inflammation by modulating pro‐inflammatory cytokines. Additionally, perilla leaf extract can suppress SARS‐CoV‐2 by inactivating the virus and reducing virus‐induced inflammation in lung cells, thereby indicating its potential for treating respiratory inflammation associated with COVID‐19 (Tang et al. 2021).

As illustrated in Figure 6E,F, exposure to PM_2.5_ significantly upregulated MUC5AC and MMP‐9 production in RPMI2650 cells. Upon exposure to PM_2.5_, the concentrations of MUC5AC and MMP‐9 in the cell lysates of untreated RPMI2650 cells (9.49 ng/mL and 50.60 pg/mL, respectively) were significantly increased by 75.44 ng/mL and 79.37 pg/mL, respectively. However, perilla leaf extracts reduced MUC5AC and MMP‐9 levels and significantly enhanced cell viability in a dose‐dependent manner, independently for the four varieties. Specifically, perilla leaf extracts of YCPL706 were most effective in reducing MUC5AC and MMP‐9 levels by 13.50 ng/mL and 45.05 pg/mL, respectively. YCPL706 effectively inhibited mucus hypersecretion and fibrosis in PM_2.5_‐induced RPMI2650 cells, demonstrating its respiratory protective and anti‐inflammatory effects. MUC5AC, an important mucin in the respiratory system, plays a crucial role in mucociliary clearance by forming a protective hydrogel barrier against pathogens. However, its dysregulation contributes to the development of various respiratory diseases. MUC5AC expression is substantially increased in patients with asthma, especially in those with cold airway hyperresponsiveness, resulting in an imbalance in mucin production that worsens disease severity (Baumlin et al. 2023). Additionally, MMPs play a crucial role in the pathogenesis and progression of various respiratory diseases, specifically those involving fibrosis. MMPs are zinc‐dependent endopeptidases that degrade extracellular matrix components, crucial for tissue remodeling and repair. However, an imbalance in MMP activity and their inhibitors can lead to pathological tissue destruction and fibrosis, as seen in diseases such as idiopathic pulmonary fibrosis, chronic pulmonary tuberculosis, and other interstitial lung diseases (Sakashita et al. 2011). The regulatory mechanisms underlying mucus secretion and MMPs are crucial in preventing respiratory diseases. No study has examined the inhibitory effect of perilla extract on inflammation, mucus hypersecretion, and fibrosis in a PM_2.5_‐exposed human nasal cell model. Additionally, there are no studies on the selection of useful resources for perilla leaves. Therefore, we identified useful GRs of perilla varieties with phenotypic diversity capable of preventing respiratory diseases. Moreover, we confirmed that YCPL706 could protect the respiratory system by reducing oxidative stress, inflammation, mucus hypersecretion, and fibrosis.

### 3.6 Antimicrobial Activities of Selected Resources

**FIGURE 8:**
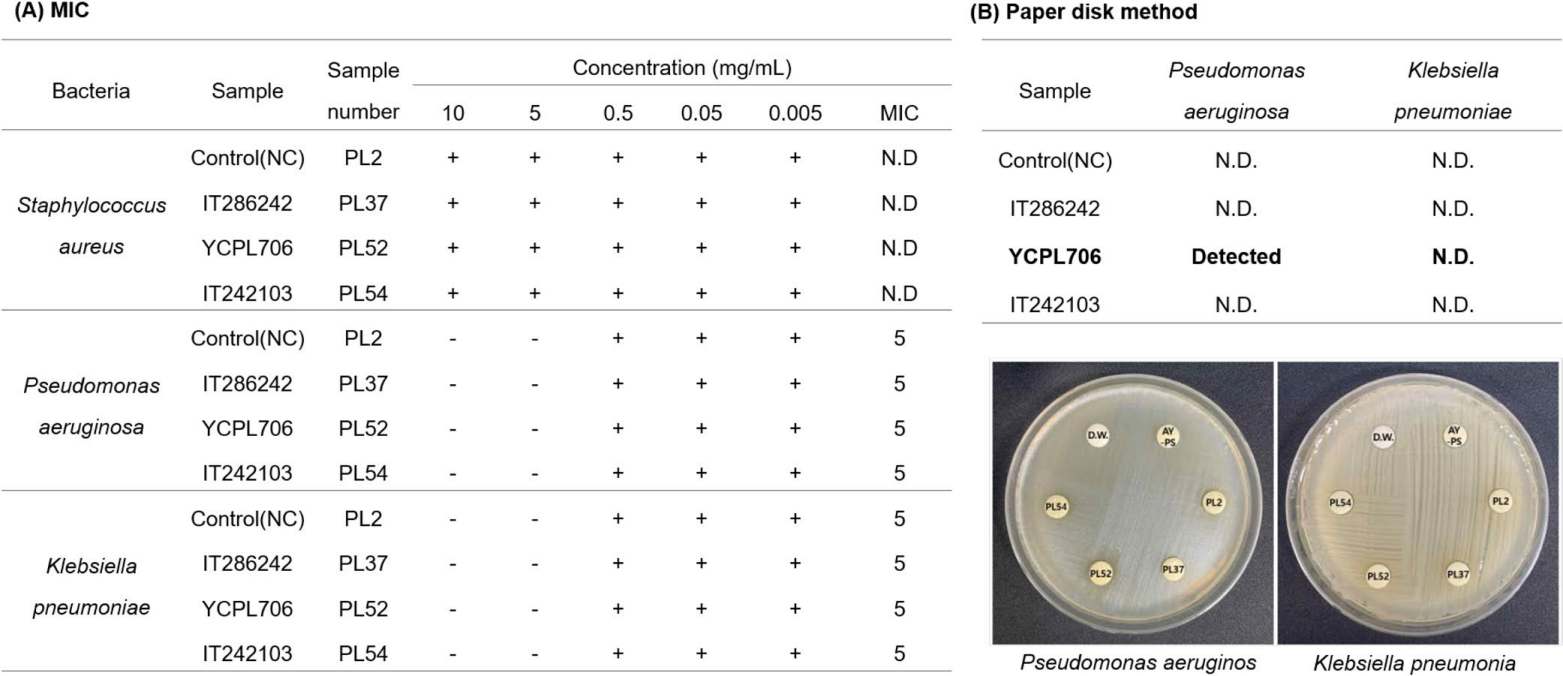
Antimicrobial activities of selected genetic resources (IT286242, YCPL706, and IT242103) compared with those of control cultivar (Namcheon) against *Staphylococcus aureus, Pseudomonas aeruginosa*, and *Klebsiella pneumoniae* . (A) Minimum inhibitory concentration (MIC) and (B) disk diffusion method. N.D.: Not detected.

Figure 8 illustrates the results of antimicrobial activities of perilla leaf extracts from selected GR (IT286242, YCPL706, and IT242103) and Namcheon against pathogens, such as *S. aureus* , *P. aeruginosa* , and *K. pneumoniae* , well‐known to be associated with respiratory diseases. These pathogens invade the lungs and are detected by the immune system. In response, immune cells like neutrophils and macrophages are activated, and these immune cells generate ROS as a defense mechanism to kill the bacteria. ROS can directly damage bacterial cell membranes, proteins, and DNA. Although ROS plays an essential role in eliminating bacterial infections in the lungs, their overproduction can contribute to lung inflammation and damage, worsening conditions like pneumonia and COPD (Riquelme and Prince 2021). Therefore, exploring natural materials that exhibit antibacterial activity against pathogens is also crucial for preventing lung inflammation and COPD. Previous studies have extensively investigated the antimicrobial effects of perilla leaves on bacteria and fungi (Belwal et al. 2016; Chen et al. 2020). The MIC values were calculated for the primary screening of antibacterial activity against the target pathogens. Treatment with the extract (5 mg/mL) inhibited *P. aeruginosa* but not *S. aureus* and *K. pneumoniae*. Additionally, the antibacterial activity of the extract (1 mg/mL) was qualitatively analyzed using the disk diffusion method to compare the activities of the control and GR against *P. aeruginosa* and *Pneumocystis carinii*. The MIC was detected and confirmed that YCPL706 exhibited clear activity, specifically against *P. aeruginosa* . In a previous study on the antimicrobial activity of perilla leaves, Ju et al. (2021) reported that aqueous ethanol extracts of *P. frutescens* var. acuta leaves, particularly the 70% and 95% ethanol extracts, exerted antimicrobial activity against *P. aeruginosa* . Additionally, He et al. (2020) reported the notable antimicrobial activity of perilla essential oil against *S. aureus* , with MIC values ranging from 1 to 2 mg/mL. Respiratory tract infections caused by *P. aeruginosa* represent a substantial challenge for clinical management owing to the pathogen's ability to form biofilms, develop intrinsic resistance to antibiotics, and evade host defenses (Rossy et al. 2022). *P. aeruginosa* , a gram‐negative bacterium, is resistant to various antibiotics and can employ epithelial cell‐secreted mucus as an energy source, inducing severe respiratory diseases. Overall, the compositions developed with YCPL706 could prevent fine dust‐caused respiratory diseases and increase and prevent respiratory diseases caused by pathogenic bacteria.

## 4 Conclusions

This study demonstrated the potential of perilla leaves ( *P. frutescens* L.) as a protective agent against PM2.5‐induced respiratory damage, highlighting the role of genetic diversity in phytochemical accumulation. Among the 56 varieties screened, three genetic resources (IT286242, YCPL706, and IT242103) showed significant respiratory protective effects by reducing key inflammation and mucus hypersecretion indicators (NO and MUC5AC). Additionally, perilla leaf extracts from YCPL706 showed notable respiratory protective effects by effectively increasing cell viability in the presence of PM_2.5_, decreasing ROS levels, and suppressing lipid peroxidation and intracellular antioxidant enzymes. Moreover, we confirmed that YCPL706 protects the respiratory system by reducing oxidative stress, inflammation, mucus hypersecretion, and fibrosis, thereby preventing respiratory diseases induced by pathogenic bacteria, such *as P. aeruginosa*. Further studies are required to elucidate the mechanism of action of the selected resource (YCPL706), identify and isolate the active compounds exerting anti‐inflammatory effects, and determine their underlying mechanism of action.

## Author Contributions

**Min Young Kim:** conceptualization (lead), investigation (lead), methodology (lead), writing – original draft (lead), writing – review and editing (lead). **Jung In Kim:** conceptualization (supporting), investigation (supporting), resources (supporting). **Sang Woo Kim:** data curation (supporting), formal analysis (lead). **Sungup Kim:** investigation (supporting), validation (supporting). **Eunyoung Oh:** methodology (supporting), validation (supporting), visualization (supporting). **Jeongeun Lee:** validation (supporting), visualization (supporting). **Eunsoo Lee:** data curation (equal). **Yeon Ju An:** data curation (supporting), visualization (supporting). **Chae‐Yeon Han:** methodology (supporting), validation (supporting). **Heungsu Lee:** supervision (supporting), writing – review and editing (supporting). **Myoung Hee Lee:** supervision (supporting), writing – review and editing (supporting).

## Ethics Statement

This study does not involve any animal or human experimentation.

## Conflicts of Interest

The authors declare no conflicts of interest.

## Supporting information


**Table S1.** Extraction yield, total polyphenol content, total flavonoid content (TFC), ABTS radical scavenging activity, DPPH radical scavenging activity, and cell viability of extracts in RPMI 2650 human nasal cell according to different resources of perilla leaves.
**Table S2.** Distribution of individual phenolic acid content (caffeic acid, rosmarinic acid, luteolin) according to different resources of perilla leaves.
**Table S3.** Distribution of cell viability (% control), nitric oxide concentration (NO, μM), and MUC5AC concentration (ng/mL) secreted in RPMI 2650 human nasal cells according to treatment with different resource extracts of perilla leaves.

## Data Availability

The data that support the findings of this study are available in the Supporting Information of this article. And, the data that support the findings of this study are available from the corresponding author upon reasonable request.
