# Supplementary material for: Respiratory Protective Effects of Perilla Leave Varieties (Perilla frutescens) Against Fine Particulate Matter (PM2.5 )‐induced Damage in Human Nasal Cells
Source: Food Sci Nutr. 2024 Dec 22;13(1):e4708. doi: 10.1002/fsn3.4708 (PMC11717003; doi:10.1002/fsn3.4708)
Supplement: Supplementary file 1 — Table S1. Extraction yield, total polyphenol content, total flavonoid content (TFC), ABTS radical scavenging activity, DPPH radical scavenging activity, and cell viability of extracts in RPMI 2650 human nasal cell according to different resources of perilla leaves. Table S2. Distribution of individual phenolic acid content (caffeic acid, rosmarinic acid, luteolin) according to different resources of perilla leaves. Table S3. Distribution of cell viability (% control), nitric oxide concentration (NO, μM), and MUC5AC concentration (ng/mL) secreted in RPMI 2650 human nasal cells according to treatment with different resource extracts of perilla leaves. [file FSN3-13-e4708-s001.docx]

**SUPPLEMENTARY MATERIAL**

**Supplementary Table S1.** Extraction yield, total polyphenol content, total flavonoid content (TFC), ABTS radical scavenging activity, DPPH radical scavenging activity, and cell viability of extracts in RPMI 2650 human nasal cell according to different resources of perilla leaves.

**Supplementary Table S2.** Distribution of individual phenolic acid content (caffeic acid, rosmarinic acid, luteolin) according to different resources of perilla leaves.

**Supplementary Table S3.** Distribution of cell viability (% control), nitric oxide concentration (NO, μM), and MUC5AC concentration (ng/mL) secreted in RPMI 2650 human nasal cells according to treatment with different resource extracts of perilla leaves.

**Supplementary Table S1.** Extraction yield, total polyphenol content, total flavonoid content (TFC), ABTS radical scavenging activity, DPPH radical scavenging activity, and cell viability of extracts in RPMI 2650 human nasal cell according to different resources of perilla leaves.

| Sample | classification | Resource | Extraction  Yield(%) | Antioxidant component | | Antioxidant activity | | Cytotoxicity |
| --- | --- | --- | --- | --- | --- | --- | --- | --- |
|  |  |  |  | TPC^1)^  (mg GAE/g) | TFC^2)^  (mg CE/g) | ABTS^3)^  (mg TE/g) | DPPH^4)^  (mg TE/g) |  |
| PL-C1 | Cultivars | Yipddeulkkae1ho | 19.78 | 30.89 ± 0.33^f^ | 20.64 ± 0.13^h^ | 58.88 ± 0.38^h^ | 57.32 ± 0.66^e^ | No (≤ 400 μg/mL) |
| PL-C2 |  | Namcheon | 19.50 | 39.05 ± 0.67^c^ | 26.30 ± 0.00^c^ | 62.58 ± 0.12^f^ | 69.87 ± 0.66^c^ | No (≤ 400 μg/mL) |
| PL-C3 |  | Donggeul 1ho | 28.21 | 60.27 ± 0.60^a^ | 41.77 ± 0.17^a^ | 91.07 ± 0.14^a^ | 81.08 ± 1.72^a^ | No (≤ 200 μg/mL) |
| PL-C4 |  | Donggeul 2ho | 18.40 | 17.22 ± 0.21^h^ | 10.37 ± 0.08^k^ | 32.89 ± 0.25^k^ | 29.20 ± 0.63^h^ | No (≤ 400 μg/mL) |
| PL-C5 |  | Soim | 21.20 | 32.34 ± 0.54^e^ | 21.23 ± 0.03^g^ | 60.79 ± 0.60^g^ | 56.83 ± 0.57^e^ | No (≤ 400 μg/mL) |
| PL-C6 |  | Sangyeup | 22.69 | 31.31 ± 0.28^f^ | 19.08 ± 0.23^i^ | 55.74 ± 0.40^i^ | 51.34 ± 0.90^f^ | No (≤ 400 μg/mL) |
| PL-C7 |  | Somirang | 20.65 | 44.67 ± 0.33^b^ | 31.86 ± 0.16^b^ | 66.52 ± 0.06^d^ | 78.13 ± 0.68^b^ | No (≤ 400 μg/mL) |
| PL-C8 |  | Saebom | 22.59 | 34.93 ± 0.77^d^ | 22.46 ± 0.01^f^ | 63.65 ± 0.54^e^ | 58.22 ± 0.80^e^ | No (≤ 400 μg/mL) |
| PL-C9 |  | Neulbora | 22.23 | 23.97 ± 0.17^g^ | 13.82 ± 0.07^j^ | 47.43 ± 0.42^j^ | 43.39 ± 1.16^g^ | No (≤ 200 μg/mL) |
| PL-C10 |  | Saebora | 22.98 | 39.53 ± 0.17^c^ | 24.46 ± 0.16^d^ | 70.26 ± 0.29^b^ | 68.15 ± 1.17^d^ | No (≤ 400 μg/mL) |
| PL-C11 |  | Bora | 21.53 | 39.04 ± 0.19^c^ | 23.00 ± 0.11^e^ | 67.69 ± 0.17^c^ | 69.21 ± 0.82^cd^ | No (≤ 200 μg/mL) |
| PL-EL1 | Elite lines | YPL147-2B-15-3-1-1-1 | 23.20 | 34.71 ± 0.40^a^ | 20.73 ± 0.01^a^ | 63.42 ± 0.73^a^ | 59.47 ± 0.63^a^ | No (≤ 400 μg/mL) |
| PL-EL2 |  | YPL151-2B-11-3-2-1-1 | 20.35 | 28.24 ± 0.44^d^ | 17.80 ± 0.01^e^ | 52.11 ± 0.14^e^ | 49.72 ± 0.37^d^ | No (≤ 400 μg/mL) |
| PL-EL3 |  | YPL167-2B-20-2-3-2-2 | 22.45 | 32.04 ± 0.21^c^ | 18.84 ± 0.09^d^ | 56.10 ± 0.41^d^ | 52.36 ± 0.58^c^ | No (≤ 400 μg/mL) |
| PL-EL4 |  | YPL166-2B-11-2-1-3-2-1 | 23.50 | 33.62 ± 0.23^b^ | 20.08 ± 0.03^b^ | 61.47 ± 0.58^b^ | 56.53 ± 0.49^b^ | No (≤ 400 μg/mL) |
| PL-EL5 |  | YPL166-2B-21-2-1-2-1-2 | 20.55 | 26.49 ± 0.17^e^ | 15.48 ± 0.10^f^ | 47.66 ± 0.47^f^ | 42.90 ± 0.75^e^ | No (≤ 400 μg/mL) |
| PL-EL6 |  | YPL172-2B-28-3-3-1-3 | 21.68 | 23.37 ± 0.22^f^ | 12.89 ± 0.11^g^ | 41.16 ± 0.81^g^ | 36.68 ± 0.78^f^ | No (≤ 400 μg/mL) |
| PL-EL7 |  | YPL175-2B-7-1-1-2-2-1 | 23.16 | 32.26 ± 0.31^c^ | 19.76 ± 0.06^c^ | 57.56 ± 0.53^c^ | 52.78 ± 0.49^c^ | No (≤ 400 μg/mL) |
| PL-GR1 | Genetic  Resource | IT105990 | 17.21 | 26.64 ± 0.37^qr^ | 16.40 ± 0.06^t^ | 48.70 ± 0.37^p^ | 45.41 ± 0.47^uv^ | No (≤ 400 μg/mL) |
| PL-GR2 |  | IT113193 | 14.87 | 20.81 ± 0.08^y^ | 12.47 ± 0.00^α^ | 37.33 ± 0.38^y^ | 34.75 ± 0.50^α^ | No (≤ 400 μg/mL) |
| PL-GR3 |  | IT117140 | 17.02 | 25.86 ± 0.25^tu^ | 15.26 ± 0.19^w^ | 46.74 ± 0.32^rs^ | 43.63 ± 0.84^x^ | No (≤ 400 μg/mL) |
| PL-GR4 |  | IT213092 | 17.51 | 27.47 ± 0.37^op^ | 16.39 ± 0.08^t^ | 49.20 ± 0.53^op^ | 46.25 ± 0.61^tu^ | No (≤ 400 μg/mL) |
| PL-GR5 |  | IT213787 | 17.34 | 33.46 ± 0.37^h^ | 20.75 ± 0.14^j^ | 55.04 ± 0.23^h^ | 57.18 ± 0.26^j^ | No (≤ 400 μg/mL) |
| PL-GR6 |  | IT223670 | 12.96 | 23.67 ± 0.44^v^ | 14.96 ± 0.05^x^ | 41.05 ± 0.30^w^ | 41.47 ± 0.62^y^ | No (≤ 400 μg/mL) |
| PL-GR7 |  | IT235791 | 18.34 | 38.11 ± 0.30^d^ | 23.69 ± 0.10^f^ | 59.18 ± 0.12^d^ | 71.64 ± 0.34^d^ | No (≤ 200 μg/mL) |
| PL-GR8 |  | IT267711 | 19.18 | 34.46 ± 0.48^fg^ | 21.12 ± 0.01^i^ | 59.31 ± 0.33^d^ | 59.22 ± 1.56^i^ | No (≤ 400 μg/mL) |
| PL-GR9 |  | IT267732 | 18.42 | 30.77 ± 0.20^k^ | 19.25 ± 0.18^n^ | 56.19 ± 0.35^g^ | 56.57 ± 0.25^jk^ | No (≤ 400 μg/mL) |
| PL-GR10 |  | IT271265 | 17.53 | 37.61 ± 0.36^de^ | 25.51 ± 0.01^d^ | 56.99 ± 0.04^f^ | 69.99 ± 0.42^e^ | No (≤ 200 μg/mL) |
| PL-GR11 |  | IT271271 | 18.84 | 34.83 ± 0.32^f^ | 19.01 ± 0.07^o^ | 58.44 ± 0.26^e^ | 63.29 ± 1.72^f^ | No (≤ 400 μg/mL) |
| PL-GR12 |  | IT271276 | 16.51 | 29.45 ± 0.57^lm^ | 17.60 ± 0.09^q^ | 51.33 ± 0.42^jk^ | 48.75 ± 0.52^pq^ | No (≤ 400 μg/mL) |
| PL-GR13 |  | IT271284 | 16.85 | 25.95 ± 0.10^su^ | 17.48 ± 0.05^qr^ | 51.41 ± 0.48^jk^ | 46.88 ± 0.41^st^ | No (≤ 400 μg/mL) |
| PL-GR14 |  | IT271292 | 17.72 | 31.41 ± 0.89^j^ | 18.97 ± 0.06^o^ | 54.92 ± 0.56^h^ | 51.19 ± 0.73^m^ | No (≤ 400 μg/mL) |
| PL-GR15 |  | IT271301 | 19.42 | 27.48 ± 0.33^op^ | 16.52 ± 0.05^t^ | 51.04 ± 0.52^jl^ | 46.80 ± 1.06^st^ | No (≤ 400 μg/mL) |
| PL-GR16 |  | IT274215 | 15.50 | 32.36 ± 0.15^i^ | 20.43 ± 0.04^k^ | 49.85 ± 0.13^no^ | 56.32 ± 0.13^jk^ | No (≤ 400 μg/mL) |
| PL-GR17 |  | IT274597 | 16.17 | 27.79 ± 0.32^o^ | 18.08 ± 0.04^p^ | 50.40 ± 0.55^ln^ | 47.45 ± 0.29^rs^ | No (≤ 400 μg/mL) |
| PL-GR18 |  | IT274644 | 14.72 | 20.28 ± 0.70^y^ | 11.93 ± 0.16^β^ | 38.42 ± 1.02^x^ | 35.23 ± 0.36^α^ | No (≤ 400 μg/mL) |
| PL-GR19 |  | IT286242 | 17.32 | 39.97 ± 0.56^c^ | 28.89 ± 0.25^b^ | 56.48 ± 0.09^fg^ | 74.30 ± 0.42^c^ | No (≤ 400 μg/mL) |
| PL-GR20 |  | IT299342 | 16.58 | 29.63 ± 0.14^lm^ | 19.55 ± 0.07^m^ | 51.57 ± 0.65^j^ | 50.17 ± 0.85^no^ | No (≤ 400 μg/mL) |
| PL-GR21 |  | IT328843 | 14.61 | 25.51 ± 0.28^u^ | 15.77 ± 0.19^v^ | 45.67 ± 0.35^t^ | 44.52 ± 0.37^vx^ | No (≤ 400 μg/mL) |
| PL-GR22 |  | YCPL32 | 14.11 | 29.29 ± 0.31^mn^ | 19.81 ± 0.01^l^ | 45.57 ± 0.10^t^ | 53.13 ± 0.35^l^ | No (≤ 400 μg/mL) |
| PL-GR23 |  | K126390 | 17.64 | 32.30 ± 0.25^i^ | 20.28 ± 0.12^k^ | 54.89 ± 0.43^h^ | 56.04 ± 0.70^k^ | No (≤ 400 μg/mL) |
| PL-GR24 |  | YCPL205-1 | 15.64 | 26.88 ± 0.33^pq^ | 17.60 ± 0.00^q^ | 49.02 ± 0.39^p^ | 47.94 ± 0.28^qr^ | No (≤ 400 μg/mL) |
| PL-GR25 |  | YCPL206-2 | 16.43 | 28.84 ± 0.94^n^ | 18.90 ± 0.09^o^ | 50.77 ± 0.36^km^ | 49.40 ± 0.15^op^ | No (≤ 400 μg/mL) |
| PL-GR26 |  | YCPL230 | 17.33 | 29.97 ± 0.42^l^ | 19.80 ± 0.13^l^ | 54.00 ± 0.43^i^ | 51.89 ± 0.40^m^ | No (≤ 400 μg/mL) |
| PL-GR27 |  | YCPL257 | 17.51 | 29.85 ± 0.25^lm^ | 19.80 ± 0.18^l^ | 54.21 ± 0.65^i^ | 51.02 ± 0.47^mn^ | No (≤ 400 μg/mL) |
| PL-GR28 |  | YCPL289 | 15.16 | 22.71 ± 0.29^w^ | 14.32 ± 0.09^y^ | 43.21 ± 0.76^u^ | 38.87 ± 0.15^z^ | No (≤ 400 μg/mL) |
| PL-GR29 |  | YCPL453 | 17.29 | 27.44 ± 0.19^op^ | 17.34 ± 0.09^r^ | 50.16 ± 0.94^mn^ | 46.24 ± 0.19^tu^ | No (≤ 400 μg/mL) |
| PL-GR30 |  | YCPL454 | 14.45 | 27.43 ± 0.23^op^ | 16.88 ± 0.05^s^ | 46.40 ± 0.14^s^ | 49.14 ± 0.37^p^ | No (≤ 400 μg/mL) |
| PL-GR31 |  | YCPL456 | 13.50 | 22.01 ± 0.11^x^ | 12.96 ± 0.05^z^ | 41.73 ± 0.36^v^ | 44.78 ± 0.59^vw^ | No (≤ 400 μg/mL) |
| PL-GR32 |  | YCPL460 | 18.04 | 33.99 ± 0.28^gh^ | 24.10 ± 0.03^e^ | 57.97 ± 0.12^e^ | 61.93 ± 0.54^g^ | No (≤ 400 μg/mL) |
| PL-GR33 |  | YCPL547 | 16.17 | 26.17 ± 0.13^rt^ | 16.17 ± 0.12^u^ | 47.11 ± 0.56^qr^ | 43.53 ± 0.14^x^ | No (≤ 400 μg/mL) |
| PL-GR34 |  | YCPL706 | 23.46 | 70.29 ± 0.48^a^ | 46.30 ± 0.23^a^ | 76.74 ± 0.07^b^ | 106.55 ± 0.18^a^ | No (≤ 200 μg/mL) |
| PL-GR35 |  | IT227027 | 19.13 | 26.50 ± 0.10^qs^ | 15.95 ± 0.16^v^ | 47.55 ± 0.55^q^ | 43.88 ± 0.57^wx^ | No (≤ 400 μg/mL) |
| PL-GR36 |  | IT242103 | 26.58 | 50.91 ± 0.67^b^ | 28.69 ± 0.20^c^ | 81.89 ± 0.46^a^ | 83.12 ± 0.74^b^ | No (≤ 200 μg/mL) |
| PL-GR37 |  | IT274280 | 24.34 | 37.18 ± 0.45^e^ | 21.45 ± 0.12^h^ | 63.67 ± 0.66^c^ | 60.60 ± 1.06^h^ | No (≤ 200 μg/mL) |
| PL-GR38 |  | YPL156-2B-9-2-1-3-2 | 22.38 | 34.23 ± 0.35^g^ | 22.46 ± 0.00^g^ | 63.50 ± 0.78^c^ | 56.84 ± 1.07^jk^ | No (≤ 400 μg/mL) |

ABTS, 2,2-azinobis (3-ethyl benzothiazoline)-6-sulfonic acid; DPPH, 1,1-diphenyl-2-picrylhydrazyl. Values are mean ± SD of three replicates. Different small letters in the same items indicate a significant difference (*p* < 0.05) between cultivars, elite lines, and genetic resources. ^1)^Total polyphenol content (mg gallic acid equivalent/g sample), ^2)^Total flavonoid content (mg catechin equivalent/g sample), ^3)^ABTS radical scavenging activity (mg Trolox equivalent/g sample), 4) DPPH radical scavenging activity (mg Trolox equivalent/g sample).

**Supplementary Table S2.** Distribution of individual phenolic acid content (caffeic acid, rosmarinic acid, luteolin) according to different resources of perilla leaves.

| Sample | classification | Resource | Phenolic compound content (mg/g) | | | |
| --- | --- | --- | --- | --- | --- | --- |
|  |  |  | Caffeic acid | Rosmarinic acid | Luteolin | Total |
| PL-C1 | Cultivars | Yipddeulkkae1ho | 0.81 ± 0.13^bd^ | 26.91 ± 3.32^cd^ | 0.09 ± 0.06^a^ | 27.81 ± 3.43^cd^ |
| PL-C2 |  | Namcheon | 0.66 ± 0.08^d^ | 34.50 ± 2.74^ab^ | 0.05 ± 0.02^b^ | 35.21 ± 2.80^ab^ |
| PL-C3 |  | Donggeul 1ho | 0.87 ± 0.26^bd^ | 36.52 ± 8.85^ab^ | 0.05 ± 0.03^b^ | 37.44 ± 9.14^ab^ |
| PL-C4 |  | Donggeul 2ho | 0.88 ± 0.10^bd^ | 38.65 ± 3.56^a^ | 0.06 ± 0.02^ab^ | 39.59 ± 3.66^a^ |
| PL-C5 |  | Soim | 0.82 ± 0.08^bd^ | 24.90 ± 1.26^cd^ | 0.02 ± 0.01^bc^ | 25.75 ± 1.33^cd^ |
| PL-C6 |  | Sangyeup | 0.95 ± 0.18^bc^ | 26.04 ± 3.75^cd^ | 0.04 ± 0.02^bc^ | 27.03 ± 3.94^cd^ |
| PL-C7 |  | Somirang | 1.23 ± 0.34^a^ | 36.61 ± 8.19^ab^ | 0.05 ± 0.03^b^ | 37.89 ± 8.55^ab^ |
| PL-C8 |  | Saebom | 0.98 ± 0.13^ab^ | 31.61 ± 2.39^ac^ | 0.02 ± 0.01^bc^ | 32.61 ± 2.48^ac^ |
| PL-C9 |  | Neulbora | 0.99 ± 0.11^ab^ | 23.85 ± 2.39^d^ | 0.02 ± 0.01^bc^ | 24.85 ± 2.50^d^ |
| PL-C10 |  | Saebora | 1.21 ± 0.16^a^ | 29.85 ± 3.09^bd^ | 0.04 ± 0.02^b^ | 31.10 ± 3.25^bd^ |
| PL-C11 |  | Bora | 0.69 ± 0.10^cd^ | 23.72 ± 2.49^d^ | ND | 24.41 ± 2.58^d^ |
| PL-EL1 | Elite lines | YPL147-2B-15-3-1-1-1 | 0.86 ± 0.12^b^ | 24.45 ± 2.51^ab^ | ND | 25.31 ± 2.62^a^ |
| PL-EL2 |  | YPL151-2B-11-3-2-1-1 | 0.72 ± 0.21^b^ | 22.56 ± 6.16^ab^ | ND | 23.29 ± 6.37^ab^ |
| PL-EL3 |  | YPL167-2B-20-2-3-2-2 | 0.73 ± 0.07^b^ | 25.89 ± 1.46^a^ | ND | 26.62 ± 1.50^a^ |
| PL-EL4 |  | YPL166-2B-11-2-1-3-2-1 | 1.02 ± 0.03^a^ | 26.71 ± 2.18^a^ | ND | 27.74 ± 2.21^a^ |
| PL-EL5 |  | YPL166-2B-21-2-1-2-1-2 | 0.80 ± 0.02^b^ | 25.32 ± 0.71^a^ | ND | 26.12 ± 0.71^a^ |
| PL-EL6 |  | YPL172-2B-28-3-3-1-3 | 0.52 ± 0.13^c^ | 19.99 ± 1.56^b^ | ND | 20.51 ± 1.54^b^ |
| PL-EL7 |  | YPL175-2B-7-1-1-2-2-1 | 0.69 ± 0.03^b^ | 23.94 ± 1.21^ab^ | ND | 24.62 ± 1.22^ab^ |
| PL-GR1 | Genetic  Resource | IT105990 | 0.72 ± 0.04^hi^ | 24.29 ± 1.21^fj^ | ND | 25.02 ± 1.25^fm^ |
| PL-GR2 |  | IT113193 | 0.93 ± 0.09^df^ | 22.10 ± 1.84^im^ | ND | 23.03 ± 1.93^jp^ |
| PL-GR3 |  | IT117140 | 0.57 ± 0.04^kl^ | 20.82 ± 1.01^km^ | ND | 21.39 ± 1.05^nq^ |
| PL-GR4 |  | IT213092 | 0.85 ± 0.12^fg^ | 25.50 ± 2.64^dg^ | ND | 26.36 ± 2.76^ci^ |
| PL-GR5 |  | IT213787 | 0.84 ± 0.05^fg^ | 29.91 ± 1.79^ab^ | ND | 30.75 ± 1.84^a^ |
| PL-GR6 |  | IT223670 | 0.49 ± 0.02^lm^ | 30.38 ± 0.50^a^ | ND | 30.88 ± 0.52^a^ |
| PL-GR7 |  | IT235791 | 1.04 ± 0.03^c^ | 23.04 ± 1.04^gk^ | ND | 24.08 ± 1.07^gn^ |
| PL-GR8 |  | IT267711 | 0.65 ± 0.05^ik^ | 24.27 ± 1.44^fj^ | ND | 24.92 ± 1.49^fm^ |
| PL-GR9 |  | IT267732 | 0.83 ± 0.10^fg^ | 23.12 ± 1.67^gk^ | ND | 23.95 ± 1.77^hn^ |
| PL-GR10 |  | IT271265 | 1.03 ± 0.06^cd^ | 24.46 ± 1.32^fi^ | 0.03 ± 0.01^e^ | 25.53 ± 1.39^ek^ |
| PL-GR11 |  | IT271271 | 0.86 ± 0.06^fg^ | 19.43 ± 0.65^mo^ | ND | 20.30 ± 0.67^pr^ |
| PL-GR12 |  | IT271276 | 0.93 ± 0.11^cf^ | 27.55 ± 3.13^be^ | ND | 28.48 ± 3.23^ad^ |
| PL-GR13 |  | IT271284 | 0.92 ± 0.13^eg^ | 28.94 ± 3.96^ac^ | ND | 29.85 ± 4.09^ab^ |
| PL-GR14 |  | IT271292 | 0.71 ± 0.11^i^ | 23.26 ± 1.39^gk^ | ND | 23.97 ± 1.50^hn^ |
| PL-GR15 |  | IT271301 | 0.63 ± 0.03^ik^ | 21.45 ± 0.95^jm^ | ND | 22.08 ± 0.98^mq^ |
| PL-GR16 |  | IT274215 | 1.00 ± 0.13^ce^ | 23.06 ± 2.31^gk^ | 0.04 ± 0.02^de^ | 24.11 ± 2.44^gn^ |
| PL-GR17 |  | IT274597 | 0.73 ± 0.05^hi^ | 26.10 ± 1.16^df^ | ND | 26.83 ± 1.20^ch^ |
| PL-GR18 |  | IT274644 | 0.20 ± 0.02^n^ | 17.00 ± 0.57^o^ | ND | 17.20 ± 0.60^s^ |
| PL-GR19 |  | IT286242 | 0.66 ± 0.05^ik^ | 27.65 ± 1.26^be^ | ND | 28.31 ± 1.29^ae^ |
| PL-GR20 |  | IT299342 | 0.94 ± 0.04^cf^ | 27.90 ± 0.80^ad^ | ND | 28.84 ± 0.83^ac^ |
| PL-GR21 |  | IT328843 | 1.26 ± 0.08^b^ | 27.04 ± 1.84^cf^ | 0.07 ± 0.02^b^ | 28.36 ± 1.93^ad^ |
| PL-GR22 |  | YCPL32 | 1.44 ± 0.02^a^ | 23.32 ± 1.12^gk^ | 0.11 ± 0.04^a^ | 24.87 ± 1.16^fm^ |
| PL-GR23 |  | K126390 | 0.59 ± 0.04^jl^ | 24.81 ± 2.14^fi^ | ND | 25.40 ± 2.18^fl^ |
| PL-GR24 |  | YCPL205-1 | 0.45 ± 0.03^m^ | 22.20 ± 0.34^im^ | ND | 22.66 ± 0.36^kq^ |
| PL-GR25 |  | YCPL206-2 | 0.46 ± 0.01^m^ | 21.44 ± 0.42^jm^ | ND | 21.90 ± 0.42^nq^ |
| PL-GR26 |  | YCPL230 | 0.64 ± 0.09^ik^ | 23.18 ± 2.87^gk^ | ND | 23.83 ± 2.95^in^ |
| PL-GR27 |  | YCPL257 | 0.42 ± 0.03^m^ | 22.05 ± 1.18^im^ | ND | 22.46 ± 1.19^lq^ |
| PL-GR28 |  | YCPL289 | 0.43 ± 0.02^m^ | 20.47 ± 0.83^km^ | ND | 20.91 ± 0.84^oq^ |
| PL-GR29 |  | YCPL453 | 0.65 ± 0.08^ik^ | 22.41 ± 1.56^hl^ | ND | 23.07 ± 1.63^jp^ |
| PL-GR30 |  | YCPL454 | 0.46 ± 0.03^m^ | 19.56 ± 1.34^mn^ | ND | 20.02 ± 1.37^qr^ |
| PL-GR31 |  | YCPL456 | 0.64 ± 0.10^ik^ | 17.48 ± 1.80^no^ | ND | 18.12 ± 1.90^rs^ |
| PL-GR32 |  | YCPL460 | 0.66 ± 0.03^ik^ | 25.08 ± 1.13^eh^ | ND | 25.74 ± 1.15^dj^ |
| PL-GR33 |  | YCPL547 | 0.44 ± 0.05^m^ | 22.68 ± 2.02^hl^ | ND | 23.12 ± 2.07^jp^ |
| PL-GR34 |  | YCPL706 | 0.99 ± 0.05^ce^ | 26.33 ± 0.72^cf^ | 0.05 ± 0.01^cd^ | 27.37 ± 0.73^bf^ |
| PL-GR35 |  | IT227027 | 0.82 ± 0.02^gh^ | 22.13 ± 0.74^im^ | 0.06 ± 0.02^bc^ | 23.01 ± 0.76^jp^ |
| PL-GR36 |  | IT242103 | 0.68 ± 0.04^ik^ | 26.25 ± 2.27^df^ | ND | 26.93 ± 2.30^cg^ |
| PL-GR37 |  | IT274280 | 0.69 ± 0.10^ij^ | 22.57 ± 1.74^hl^ | ND | 23.26 ± 1.84^jo^ |
| PL-GR38 |  | YPL156-2B-9-2-1-3-2 | 0.58 ± 0.06^kl^ | 19.91 ± 1.38^ln^ | ND | 20.48 ± 1.44^or^ |

Values are mean ± SD of three replicates. Different small letters in the same items indicate a significant difference (*p < 0.05*) between cultivars, elite lines, and genetic resources.

**Supplementary Table S3.** Distribution of cell viability (% control), nitric oxide concentration (NO, μM), and MUC5AC concentration (ng/mL) secreted in RPMI 2650 human nasal cells according to treatment with different resource extracts of perilla leaves.

| PM 2.5 | Sample | classification | Resource | Cell viability (% Control) | NO concentration (μM) | MUC5AC concentration (ng/mL) |
| --- | --- | --- | --- | --- | --- | --- |
|  |  |  |  |  |  |  |
| - | - | Control | | 100.00 ± 2.29 | 6.74 ± 1.39 | 10.28 ± 0.79 |
| + | - |  |  | 46.48 ± 1.16^***^ | 41.03 ± 1.27^***^ | 64.63 ± 1.03*** |
| + | PL-C1 | Cultivars | Yipddeulkkae1ho | 84.18 ± 0.20^c^ | 27.24 ± 2.53^de^ | 38.05 ± 1.25^ab^ |
| + | PL-C2 |  | Namcheon | 82.15 ± 0.82^cd^ | 29.98 ± 1.57^cd^ | 36.13 ± 3.34^a^ |
| + | PL-C3 |  | Donggeul 1ho | 80.21 ± 3.98^d^ | 33.63 ± 0.41^ab^ | 41.29 ± 1.53^b^ |
| + | PL-C4 |  | Donggeul 2ho | 69.25 ± 1.23^f^ | 15.46 ± 2.72^f^ | 54.88 ± 2.69^de^ |
| + | PL-C5 |  | Soim | 72.24 ± 1.16^ef^ | 15.89 ± 0.97^f^ | 61.36 ± 1.22^f^ |
| + | PL-C6 |  | Sangyeup | 75.22 ± 1.01^e^ | 16.22 ± 2.33^f^ | 56.31 ± 1.38^e^ |
| + | PL-C7 |  | Somirang | 94.21 ± 2.02^b^ | 26.43 ± 0.89^e^ | 50.78 ± 5.61^cd^ |
| + | PL-C8 |  | Saebom | 101.15 ± 3.61^a^ | 28.31 ± 1.30^ce^ | 47.89 ± 3.17^c^ |
| + | PL-C9 |  | Neulbora | 84.22 ± 2.04^c^ | 31.59 ± 1.81^bc^ | 46.28 ± 1.29^c^ |
| + | PL-C10 |  | Saebora | 95.16 ± 1.68^b^ | 35.57 ± 1.59^a^ | 72.20 ± 2.94^g^ |
| + | PL-C11 |  | Bora | 92.16 ± 0.52^b^ | 15.52 ± 3.17^f^ | 54.82 ± 2.38^de^ |
| + | PL-EL1 | Elite lines | YPL147-2B-15-3-1-1-1 | 91.17 ± 1.32^a^ | 7.99 ± 2.69^f^ | 61.05 ± 1.82^d^ |
| + | PL-EL2 |  | YPL151-2B-11-3-2-1-1 | 75.21 ± 2.02^d^ | 31.11 ± 0.92^a^ | 51.27 ± 1.68^c^ |
| + | PL-EL3 |  | YPL167-2B-20-2-3-2-2 | 78.23 ± 0.89^c^ | 26.54 ± 1.05^b^ | 50.68 ± 7.19^c^ |
| + | PL-EL4 |  | YPL166-2B-11-2-1-3-2-1 | 81.40 ± 1.00^b^ | 17.18 ± 0.34^de^ | 47.65 ± 3.65^bc^ |
| + | PL-EL5 |  | YPL166-2B-21-2-1-2-1-2 | 72.19 ± 1.83^e^ | 15.14 ± 0.73^e^ | 43.94 ± 0.94^ab^ |
| + | PL-EL6 |  | YPL172-2B-28-3-3-1-3 | 68.47 ± 2.16^f^ | 22.83 ± 1.46^c^ | 38.71 ± 1.96^a^ |
| + | PL-EL7 |  | YPL175-2B-7-1-1-2-2-1 | 81.17 ± 0.63^b^ | 18.85 ± 1.50^d^ | 72.38 ± 2.76^e^ |
| + | PL-GR1 | Genetic  Resource | IT105990 | 72.24 ± 0.85^no^ | 25.14 ± 1.29^cf^ | 62.35 ± 1.86^ik^ |
| + | PL-GR2 |  | IT113193 | 69.23 ± 1.41^op^ | 24.39 ± 0.97^df^ | 54.78 ± 1.99^gh^ |
| + | PL-GR3 |  | IT117140 | 79.24 ± 4.69^m^ | 29.87 ± 2.22^a^ | 46.54 ± 7.57^df^ |
| + | PL-GR4 |  | IT213092 | 75.19 ± 0.94^n^ | 27.72 ± 1.50^b^ | 42.95 ± 2.13^ce^ |
| + | PL-GR5 |  | IT213787 | 92.30 ± 1.62^eg^ | 12.51 ± 2.94^no^ | 40.51 ± 6.52^bd^ |
| + | PL-GR6 |  | IT223670 | 81.26 ± 1.68^lm^ | 14.23 ± 1.06^ln^ | 70.86 ± 4.07^ln^ |
| + | PL-GR7 |  | IT235791 | 95.24 ± 2.78^ef^ | 14.98 ± 0.73^lm^ | 35.20 ± 2.60^b^ |
| + | PL-GR8 |  | IT267711 | 87.16 ± 2.04^hj^ | 25.30 ± 1.21^ce^ | 58.61 ± 0.79^hj^ |
| + | PL-GR9 |  | IT267732 | 86.22 ± 2.59^ij^ | 24.92 ± 0.97^cf^ | 62.89 ± 1.01^ik^ |
| + | PL-GR10 |  | IT271265 | 95.17 ± 2.72^ef^ | 22.83 ± 1.05^fh^ | 54.78 ± 0.54^gh^ |
| + | PL-GR11 |  | IT271271 | 90.18 ± 3.61^gh^ | 27.08 ± 1.55^bc^ | 59.54 ± 2.58^hj^ |
| + | PL-GR12 |  | IT271276 | 96.21 ± 2.01^de^ | 25.03 ± 0.90^cf^ | 62.69 ± 4.56^ik^ |
| + | PL-GR13 |  | IT271284 | 95.18 ± 1.05^ef^ | 21.65 ± 0.97^gi^ | 56.49 ± 3.97^gi^ |
| + | PL-GR14 |  | IT271292 | 90.16 ± 3.99^gh^ | 23.63 ± 3.31^dg^ | 76.60 ± 1.30^no^ |
| + | PL-GR15 |  | IT271301 | 92.48 ± 0.58^eg^ | 22.72 ± 0.81^fh^ | 62.00 ± 2.48^ij^ |
| + | PL-GR16 |  | IT274215 | 91.25 ± 1.12^fg^ | 11.86 ± 1.04^op^ | 54.45 ± 8.86^gh^ |
| + | PL-GR17 |  | IT274597 | 82.25 ± 2.60^km^ | 20.89 ± 0.73^hi^ | 36.71 ± 2.54^bc^ |
| + | PL-GR18 |  | IT274644 | 70.30 ± 2.84^op^ | 25.03 ± 1.77^cf^ | 41.78 ± 3.75^ce^ |
| + | PL-GR19 |  | IT286242 | 102.5 ± 1.81^c^ | 11.54 ± 1.07^op^ | 73.75 ± 3.06^mo^ |
| + | PL-GR20 |  | IT299342 | 80.21 ± 1.84^m^ | 19.92 ± 1.34^i^ | 60.85 ± 0.68^hj^ |
| + | PL-GR21 |  | IT328843 | 75.16 ± 1.32^n^ | 21.65 ± 0.90^gi^ | 51.81 ± 3.34^fg^ |
| + | PL-GR22 |  | YCPL32 | 92.52 ± 0.56^eg^ | 15.73 ± 0.65^kl^ | 46.91 ± 7.57^ef^ |
| + | PL-GR23 |  | K126390 | 90.23 ± 1.10^gh^ | 17.72 ± 1.07^jk^ | 61.88 ± 2.56^ij^ |
| + | PL-GR24 |  | YCPL205-1 | 72.20 ± 1.90^no^ | 23.58 ± 1.77^dg^ | 62.67 ± 0.76^ik^ |
| + | PL-GR25 |  | YCPL206-2 | 73.07 ± 1.03^no^ | 22.88 ± 0.49^eh^ | 63.38 ± 0.78^ik^ |
| + | PL-GR26 |  | YCPL230 | 65.19 ± 0.91^q^ | 21.32 ± 0.74^gi^ | 59.88 ± 0.83^hj^ |
| + | PL-GR27 |  | YCPL257 | 68.05 ± 0.95^pq^ | 25.78 ± 0.97^bd^ | 36.79 ± 3.02^bc^ |
| + | PL-GR28 |  | YCPL289 | 72.17 ± 1.82^no^ | 23.69 ± 0.49^dg^ | 23.27 ± 1.16^a^ |
| + | PL-GR29 |  | YCPL453 | 79.25 ± 1.28^m^ | 22.88 ± 0.65^eh^ | 21.66 ± 2.89^a^ |
| + | PL-GR30 |  | YCPL454 | 92.18 ± 1.49^eg^ | 15.84 ± 0.48^kl^ | 38.55 ± 2.91^bc^ |
| + | PL-GR31 |  | YCPL456 | 82.19 ± 0.73^km^ | 21.54 ± 0.61^gi^ | 50.68 ± 2.46^fg^ |
| + | PL-GR32 |  | YCPL460 | 84.16 ± 1.13^jl^ | 20.89 ± 0.65^hi^ | 61.80 ± 2.48^ij^ |
| + | PL-GR33 |  | YCPL547 | 85.16 ± 0.55^jk^ | 14.17 ± 0.99^ln^ | 62.89 ± 2.30^ik^ |
| + | PL-GR34 |  | YCPL706 | 112.48 ± 2.65^a^ | 9.98 ± 0.65^pq^ | 78.65 ± 3.32^o^ |
| + | PL-GR35 |  | IT227027 | 89.20 ± 3.21^gi^ | 12.83 ± 0.67^mo^ | 60.79 ± 2.81^hj^ |
| + | PL-GR36 |  | IT242103 | 107.34 ± 2.70^b^ | 8.90 ± 0.65^q^ | 77.42 ± 3.47^o^ |
| + | PL-GR37 |  | IT274280 | 101.22 ± 2.12^c^ | 15.03 ± 0.74^lm^ | 68.72 ± 3.08^km^ |
| + | PL-GR38 |  | YPL156-2B-9-2-1-3-2 | 99.22 ± 3.41^cd^ | 19.66 ± 1.23^ij^ | 64.95 ± 3.34^jl^ |

Values are mean ± SD of three replicates. Different small letters in the same items indicate a significant difference (*p < 0.05*) between cultivars, elite lines, and genetic resources.
